# (Pre)diabetes, glycemia, and daily glucose variability are associated with retinal nerve fiber layer thickness in The Maastricht Study

**DOI:** 10.1038/s41598-022-22748-2

**Published:** 2022-10-22

**Authors:** Frank C. T. van der Heide, Yuri D. Foreman, Iris W. M. Franken, Ronald M. A. Henry, Abraham A. Kroon, Pieter C. Dagnelie, Simone J. P. M. Eussen, Tos T. J. M. Berendschot, Jan S. A. G. Schouten, Carroll A. B. Webers, Miranda T. Schram, Carla J. H. van der Kallen, Marleen M. J. van Greevenbroek, Anke Wesselius, Casper G. Schalkwijk, Nicolaas C. Schaper, Martijn C. G. J. Brouwers, Coen D. A. Stehouwer

**Affiliations:** 1 CARIM School for Cardiovascular Diseases, Maastricht University (UM), Maastricht, The Netherlands; 2 Department of Internal Medicine, Maastricht University Medical Center+ (MUMC+), P. Debyelaan 25, P.O. Box 5800, 6202AZ Maastricht, The Netherlands; 3 Heart and Vascular Center, MUMC+ Maastricht, Maastricht, The Netherlands; 4 Department of Epidemiology, UM, Maastricht, The Netherlands; 5 University Eye Clinic Maastricht, MUMC+, Maastricht, The Netherlands; 6 Department of Ophthalmology, Canisius Wilhelmina Hospital, Nijmegen, The Netherlands; 7 Department of Complex Genetics and Epidemiology, NUTRIM School for Nutrition and Translational Research in Metabolism, UM, Maastricht, The Netherlands; 8 CAPHRI Care and Public Health Research Institute, UM, Maastricht, The Netherlands; 9 Division of Endocrinology and Metabolic Disease, Department of Internal Medicine, Maastricht University Medical Centre+, Maastricht, The Netherlands

**Keywords:** Biomarkers, Diseases, Endocrinology, Medical research, Neurology, Risk factors

## Abstract

Retinopathy and neuropathy in type 2 diabetes are preceded by retinal nerve fibre layer (RNFL) thinning, an index of neurodegeneration. We investigated whether glucose metabolism status (GMS), measures of glycaemia, and daily glucose variability (GV) are associated with RNFL thickness over the entire range of glucose tolerance. We used cross-sectional data from The Maastricht Study (up to 5455 participants, 48.9% men, mean age 59.5 years and 22.7% with type 2 diabetes) to investigate the associations of GMS, measures of glycaemia (fasting plasma glucose [FPG], 2-h post-load glucose [2-h PG], HbA1c, advanced glycation endproducts [AGEs] assessed as skin autofluorescence [SAF]) and indices of daily GV (incremental glucose peak [IGP] and continuous glucose monitoring [CGM]-assessed standard deviation [SD]) with mean RNFL thickness. We used linear regression analyses and, for GMS, P for trend analyses. We adjusted associations for demographic, cardiovascular risk and lifestyle factors, and, only for measures of GV, for indices of mean glycaemia. After full adjustment, type 2 diabetes and prediabetes (versus normal glucose metabolism) were associated with lower RNFL thickness (standardized beta [95% CI], respectively − 0.16 [− 0.25; − 0.08]; − 0.05 [− 0.13;  0.03]; P_trend_ = 0.001). Greater FPG, 2-h PG, HbA1c, SAF, IGP, but not CGM-assessed SD, were also associated with lower RNFL thickness (per SD, respectively − 0.05 [− 0.08; − 0.01]; − 0.06 [− 0.09; − 0.02]; − 0.05 [− 0.08; − 0.02]; − 0.04 [− 0.07; − 0.01]; − 0.06 [− 0.12; − 0.01]; and − 0.07 [− 0.21; 0.07]). In this population-based study, a more adverse GMS and, over the entire range of glucose tolerance, greater glycaemia and daily GV were associated with lower RNFL thickness. Hence, early identification of individuals with hyperglycaemia, early glucose-lowering treatment, and early monitoring of daily GV may contribute to the prevention of RNFL thinning, an index of neurodegeneration and precursor of retinopathy and neuropathy.

## Introduction

Retinopathy and neuropathy, both hallmark microvascular complications of type 2 diabetes, are preceded by subtle neurodegenerative changes. Such changes include retinal nerve fibre layer (RNFL) thinning, which can be non-invasively assessed^1–3^. RNFL thinning reflects a gradual loss of retinal ganglion cell axons, which transmit visual information from the retina to the brain^1,2^. Mechanistically, elevated glucose concentrations are thought to be toxic for retinal ganglion cells as well as for retinal endothelial and glia cells, which contribute to local metabolic regulation^4,5^. Thus, hyperglycaemia can lead to retinal ganglion cell apoptosis and loss of retinal ganglion cell axons both directly and through impairment of endothelial and glia cell function^6^.

As postulated in the “ticking clock hypothesis”, hyperglycaemia-mediated damage is thought to be a continuous (i.e., linear) process that already starts before the onset of type 2 diabetes^7,8^. Indeed, our group observed linear associations of more adverse glucose metabolism status (GMS) and higher glycaemia (estimated by various measures) with lower heart rate variability^9^, more structural brain abnormalities^10,11^, and worse peripheral nerve function^12^, all of which are measures of neurodegeneration.

The current literature on the associations of hyperglycaemia with RNFL thickness has some important limitations. First, in previous population-based studies, several important confounders were not included, such as age^13–15^, sex^14,15^, socioeconomic status^13–23^, cardiovascular risk factors^13–15,19,21–24^, and lifestyle factors (e.g., alcohol use^13,16–19,21^, diet^13–24^, and physical activity)^13–19,21–24^. Second, no population-based studies have yet investigated whether advanced glycation endproducts (AGEs) or daily glucose variability are associated with RNFL thickness. It is important to establish to what extent hyperglycaemia (including AGEs) and daily glucose variability contribute to RNFL thinning over the entire range of glucose tolerance because early diagnosis and treatment of hyperglycaemia as well as novel strategies to monitor glycaemic exposure may contribute to the early prevention of hyperglycaemia-mediated RNFL thinning^5,25,26^.

In view of above, we investigated, using a large, well-characterized population-based cohort study, whether more adverse GMS, greater glycaemia, and greater daily glucose variability are associated with lower RNFL thickness.

## Methods

### Study population and design

We used data from The Maastricht Study, a prospectively designed, population-based observational cohort study. The rationale and methodology have been described previously^27^. In brief, the study focuses on the aetiology, pathophysiology, complications and comorbidities of type 2 diabetes mellitus and is characterized by an extensive phenotyping approach. Eligible for participation were all individuals aged between 40 and 75 years and living in the southern part of the Netherlands. Participants were recruited through mass media campaigns and from the municipal registries and the regional Diabetes Patient Registry via mailings. Recruitment was stratified according to known type 2 diabetes status, with an oversampling of individuals with type 2 diabetes, for reasons of efficiency^27^. The present report includes cross-sectional data of 8005 participants who completed the baseline survey between November 2010 and September 2018. The examinations of each participant were performed within a time window of three months. From 19 September 2016 until 13 September 2018, participants were invited to also undergo CGM^26^. During this period, a selected group of recently included participants was invited to return for CGM (‘catch-up visit’). For these participants only there was a median time interval (‘visit interval’) of 2.1 years between CGM and all other measurements (more details are provided in the Supplemental Material).

### Glucose metabolism status

After an overnight fast, participants underwent a standardized seven-point oral glucose tolerance test (OGTT) as part of which venous samples were collected at 15, 30, 45, 60, 90, and 120 min post ingestion of a 75 g glucose drink. All participants underwent an OGTT except for the participants who used insulin or had a fasting plasma glucose (FPG) concentration above 11.0 mmol/L. Based on FPG and 2-h post-load glucose, GMS was determined as normal glucose metabolism (NGM), prediabetes (impaired fasting glucose, impaired glucose tolerance, or both), type 2 diabetes, or other types of diabetes (including type 1 diabetes) in accordance with the World Health Organization 2006 criteria^28^.

### Measures of glycaemia

FPG (mmol/L) and haemoglobin A1c (HbA1c; mmol/mol; %) were determined in venous plasma samples collected after an overnight fast. Two-hour post-load glucose (mmol/L) was determined in venous plasma collected at 120 min post glucose drink ingestion. AGEs were assessed with the AGE Reader (DiagnOptics Technologies BV, Groningen, the Netherlands). In brief, the AGE Reader is a desktop device that uses the characteristic fluorescent properties of certain AGEs to quantify their accumulation in the skin as skin autofluorescence (SAF; arbitrary units [AU])^29^. The AGE Reader illuminates a skin surface of 4 cm^2^, shielded from other light, and uses the ratio of the reflection of fluorescent light (wavelength 420 to 600 nm) to non-fluorescent light (300–420 nm) to calculate SAF (more details are provided in the Supplemental Materials).

### Indices of daily glucose variability

The incremental glucose peak (IGP; mmol/L), a recently validated OGTT-based index of daily glucose variability, was calculated by subtracting FPG from the maximum glucose peak value measured during a complete seven-point OGTT^30^. At the time of analysis, data on IGP were available in a subset of participants (n = 2407). We used CGM (iPro2 and Enlite Glucose Sensor; Medtronic, Tolochenaz, Switzerland) to assess daily glucose variability during a 1-week period, which was expressed as standard deviation (mmol/L) of mean sensor glucose (mmol/L)^26^. CGM-assessed data were available in a subset of participants (n = 622). More details on the assessment of glycemic indices with OGTT and CGM are provided in the Supplemental Methods.

### RNFL thickness

We assessed peripapillary RNFL thickness (μm) in both eyes using optical coherence tomography (OCT) (Spectralis unit and Eye Explorer version 5.7.5.0 software; Heidelberg Engineering, Heidelberg, Germany; 3.45-mm-diameter circle scan, manually centred on optic nerve head, 12°, 768 voxels, 100 automatic real-time tracking). Intra- and interindividual reliability, expressed as intraclass correlation coefficients, are 0.97 and 0.96 respectively^31^. At least 15 min before the examination, pupils were dilated with topical 0.5% tropicamide and 2.5% phenylephrine. Experienced graders masked to clinical information on the participants reviewed the OCT scans and graded their quality. OCT images were excluded if one of the following criteria was present: scan error (i.e., incomplete scan, poor centring of the circular scan on the optic nerve head, RNFL layer incorrectly defined, or technical problem with the OCT device) or poor imaging quality (signal-to-noise ratio < 15 dB)^23^. If data from both eyes were available (n = 2796 participants) we averaged RNFL thickness in order to reduce measurement error. If data from only one eye were available (n = 2755 participants), we used the RNFL thickness of that eye in the analyses. More details, including on quality criteria, are shown in the Supplemental Methods.

### Covariates

As described previously^27^, we assessed educational level (low, intermediate, high), socio-economic status (income level and occupational status) ^32^, smoking status (never, former, current), alcohol use (none, low, high), history of cardiovascular disease, and duration of diabetes by questionnaire; assessed dietary habits (“dietary intake”) with the Dutch Healthy Diet index sum score, a measure of adherence to the Dutch dietary guidelines 2015^33^, based on a validated food frequency questionnaire^34^; assessed lipid-modifying, antihypertensive, intraocular pressure-lowering, and glucose-lowering medication use as part of a medication interview; assessed weight, height, and waist circumference during a physical examination; calculated body mass index (BMI) based on body weight and height; measured office and 24-h ambulatory blood pressure (BP); measured total daily physical activity (hours/day) with an accelerometer^35^; measured lipid profile and plasma biomarkers of low-grade inflammation^36^ (i.e., high-sensitive C-reactive protein, serum amyloid A, interleukin-6, interleukin-8 and tumour necrosis factor alpha) in fasting venous blood samples; measured urinary albumin excretion in two 24-h urine collections; calculated the estimated glomerular filtration rate (eGFR) based on serum creatinine only, since cystatin C was not presently available in all study participants; assessed presence of retinopathy in both eyes via fundus photography (more details in the Supplemental Material); and used an automated refractor and noncontact tonometer (Tonoref II; Nidek, Gamagordi, Japan) to assess spherical equivalent and intraocular pressure in both eyes. Glaucoma was defined as use of intraocular pressure-lowering medication, intraocular pressure higher than 21 mm Hg in any eye (91.3% of all participants had data on intraocular pressure available for at least 1 eye), or both. Spherical equivalent was defined as the mean spherical equivalent of both eyes (available for 91.1% of all participants) or as the spherical equivalent of the eye for which data were available.

### Statistical analysis

We used multivariable linear regression analyses to investigate the associations of GMS (entered as dummy variables of prediabetes, type 2 diabetes, or other types of diabetes versus NGM) and standardized FPG, 2-h post-load glucose, HbA1c, SAF, IGP, and CGM-assessed standard deviation (determinants) with standardized mean RNFL thickness (outcome).

We performed P for trend analyses to test for linear trend with more adverse GMS. To test for trend, we entered GMS into the model as an ordinal variable (i.e., GMS was coded as NGM = 0, prediabetes = 1, type 2 diabetes = 2).In P for trend analyses we excluded participants with other types of diabetes because other types of diabetes (such as type 1 diabetes) do not constitute part of the spectrum of deterioration of GMS from NGM to prediabetes and type 2 diabetes. Then, we checked whether we could assume a linear trend by comparing the statistical variance explained by the statistical model in which GMS was entered as dummy variables to the statistical model in which GMS was entered as an ordinal variable. We used a likelihood ratio test to assess whether the amount of variance explained by both models differed statistically significantly. A P-value > 0.05 indicates that both models are not different and, thus, that a linear trend can be assumed. For all analyses under study, the P-value for the likelihood ratio test was > 0.05 (data not shown) and therefore a linear trend could be assumed.

Model 1 shows crude results. In model 2, we adjusted for age, sex, educational status (low, medium, high). We chose these variables because they are key potential confounders^17^. In model 3, we additionally adjusted for variables of which their status as confounder has been less firmly established (office systolic BP, use of antihypertensive medication [yes/no], waist circumference, total cholesterol / HDL cholesterol ratio, lipid-modifying medication [yes/no], smoking status [current, former, never], and alcohol consumption status [none, low, high])^17^. Then, and only for IGP and CGM-assessed standard deviation, we additionally adjusted for HbA1c or mean sensor glucose, respectively (model 3 + HbA1c; model 3 + mean sensor glucose), so that we could differentiate between daily glucose variability and average glycaemia, both of which are strongly related^37^. Additionally, and only for CGM-assessed indices, we entered ‘visit interval’ in model 1. The associations were expressed as standardized regression coefficient (stβ) and corresponding 95% confidence interval (95%CI). Collinearity diagnostics (i.e., tolerance < 0.10 and/or variance inflation factor > 10) were used to detect excessive multicollinearity between covariates.

We tested for interaction to assess whether associations differed by GMS (i.e., between individuals with type 2 diabetes, individuals with prediabetes, and individuals with NGM) or by sex. For interaction analyses with GMS, we excluded participants with other types of diabetes from the interaction analyses because the number of these participants was small.

To assess the robustness of our findings we performed several sensitivity analyses. In brief, we adjusted for potential confounders which we did not include in the main model (e.g. for lifestyle factors [dietary intake, physical activity] or ocular variables [spherical equivalent, intraocular pressure])^38–41^; we performed analyses with other indices of CGM-assessed daily glucose variability that were not included in the main manuscript (i.e. coefficient of variation [CV], time-in-range [TIR], time below range [TBR]; and time above range [TAR])^42,43^; and we repeated analyses after replacement of covariates which other covariates which reflect a similar underlying construct (e.g. we replaced waist circumference with BMI). A detailed overview of all sensitivity analyses is presented in the Supplemental Methods section.

All analyses were performed with Statistical Package for Social Sciences version 25.0 (IBM SPSS, IBM Corp, Armonk, NY, USA). For all analyses, a P-value < 0.05 was considered statistically significant.

### Ethics declaration

The study has been approved by the institutional medical ethical committee (NL31329.068.10) and the Minister of Health, Welfare and Sports of the Netherlands (Permit 131088-105234-PG). All participants gave written informed consent^27^.

## Results

### Selection and characteristics of the study population

**Figure 1:**
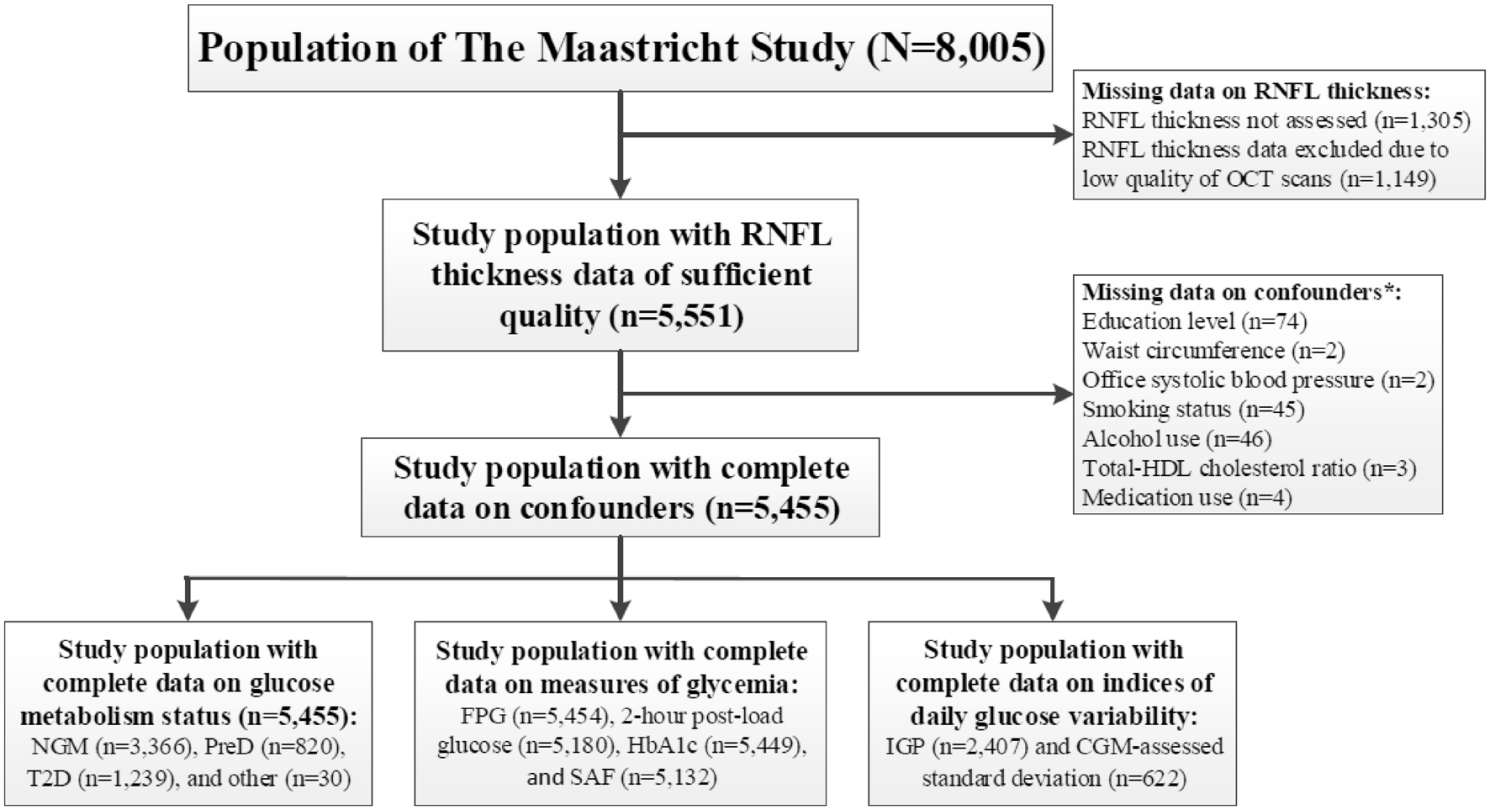
RNFL study population selection. * Not mutually exclusive. OCT, optical coherence tomography; RNFL, retinal nerve fiber layer; HDL, high density lipoprotein; NGM, normal glucose metabolism; PreD, prediabetes; T2D, type 2 diabetes; FPG, fasting plasma glucose; HbA1c, hemoglobin A1C; SAF, skin autofluorescence; IGP, incremental glucose peak; CGM, continuous glucose monitoring.

Figure 1 gives an overview of the study population selection. Participants in whom OCT data were missing or of insufficient quality were excluded first (n = 2454). Next, individuals with missing data on confounders were excluded (n = 96). The sample size of the final study populations depended on the availability of data on the main determinant (n = 5455 for GMS, n = 982 to 5454 for measures of glycaemia, and n = 622 to 2407 for indices of daily glucose variability).

Table 1 and Supplemental Table 1 show general participant characteristics according to tertiles of RNFL thickness. Overall, participants with a thinner RNFL were more often men, were older, and had a more adverse cardiovascular risk profile. General characteristics of participants included in the study were comparable to those of participants with missing data (Supplemental Table S2).

**Table 1:** General study population characteristics according to tertiles of the retinal nerve fiber layer thickness in the study population with complete data on glucose metabolism status.

	RNFL thickness			
	Total study population (n = 5455)	Tertile 1 (high) (n = 1818)	Tertile 2 (middle) (n = 1819)	Tertile 3 (low) (n = 1818)
**Characteristic**				
Age (years)	59.5 ± 8.6	59.1 ± 8.7	59.3 ± 8.7	60.0 ± 8.5
Men	2665 (48.9)	805 (48.9)	847 (46.6)	1013 (55.7)
**Educational level**				
Low	1914 (35.1)	669 (36.8)	651 (35.8)	594 (32.7)
Medium	1519 (27.8)	525 (28.9)	505 (27.8)	489 (26.9)
High	2022 (37.1)	624 (34.3)	663 (36.4)	735 (40.4)
**Glucose metabolism status**				
NGM	3366 (61.7)	1174 (64.6)	1144 (62.9)	1048 (57.6)
Prediabetes	820 (15.0)	266 (14.6)	280 (15.4)	274 (15.1)
Type 2 diabetes	1239 (22.7)	370 (20.4)	383 (21.1)	486 (26.7)
Other type of diabetes	30 (0.5)	8 (0.4)	12 (0.7)	10 (0.6)
**Measures of glycaemia**				
Fasting plasma glucose (mmol/L)*	5.9 ± 1.5	5.8 ± 1.4	5.8 ± 1.5	6.0 ± 1.7
2-h post-load glucose (mmol/L)*	6.2 [4.9–8.6]	6.1 [4.9–8.2]	6.1 [4.9–8.5]	6.3 [5.1–9.2]
HbA1c (mmol/mol)*	39.2 ± 9.1	38.7 ± 8.5	39.1 ± 9.3	39.9 ± 9.5
HbA1c (%)*	5.7 ± 0.8	5.7 ± 0.8	5.7 ± 0.8	5.8 ± 0.9
Skin autofluorescence (AU)*	2.2 ± 0.5	2.1 ± 0.5	2.2 ± 0.5	2.2 ± 0.5
**Indices of daily glucose variability**				
Incremental glucose peak (mmol/L)*	4.1 [2.7–6.5]	3.9 [2.6–5.9]	4.1 [2.8–6.5]	4.4 [2.8–7.0]
CGM-assessed standard deviation (mmol/L)*	0.86 [0.68–1.21]	0.86 [0.67–1.18]	0.85 [0.67–1.17]	0.88 [0.70–1.29]
Use of glucose-modifying medication, yes vs. no	927 (17.0)	266 (14.6)	289 (15.9)	372 (20.5)
Alfaglucosidase inhibitor, yes vs. no	0 (0.0)	0 (0.0)	0 (0.0)	0 (0.0)
Biguanides, yes vs. no	796 (14.6)	224 (12.4)	254 (14.0)	317 (15.4)
DPP4 inhibitor, yes vs. no	71 (1.5)	21 (1.2)	21 (1.2)	29 (2.1)
GLP-1 analoges, yes vs. no	10 (0.2)	5 (0.3)	1 (0.1)	4 (0.2)
SGLT2 nhibitor, yes vs. no	2 (< 0.1)	1 (0.1)	1 (0.1)	0 (0.0)
Sulfonylureumderivates, yes vs. no	226 (4.1)	62 (3.4)	70 (3.8)	94 (5.2)
Thiazolidinedion, yes vs. no	12 (0.2)	3 (0.2)	1 (0.1)	8 (0.4)
Insulin, yes vs. no	233 (4.3)	71 (3.9)	69 (3.8)	93 (5.1)
Waist circumference, men (cm)	100.4 ± 11.6	100.2 ± 11.7	99.9 ± 11.7	101.0 ± 11.5
Waist circumference, women (cm)	89.1 ± 12.5	89.2 ± 12.8	88.9 ± 12.1	89.2 ± 12.8
Total-to-HDL cholesterol ratio	3.4 [2.8–4.2]	3.3 [2.7–4.1]	3.3 [2.8–4.2]	3.4 [2.8–4.2]
Use of lipid-modifying medication, yes vs. no	1687 (30.9)	535 (29.4)	541 (29.7)	611 (33.6)
Office systolic blood pressure (mmHg)	133.2 ± 17.7	132.0 ± 18.0	133.0 ± 17.6	134.5 ± 17.5
Office diastolic blood pressure (mmHg)	75.5 ± 9.8	75.0 ± 9.8	75.3 ± 9.7	76.4 ± 9.9
Use of antihypertensive medication, yes vs. no	1983 (36.4)	601 (33.1)	637 (35.0)	745 (41.0)
Retinopathy**	79 (1.5)	8 (1.3)	5 (0.8)	12 (1.7)
Spherical equivalent (diopter)	0.13 [− 1.19–1.06]	0.6 [− 0.4–1.6]	0.1 [− 1.1–1.1]	− 0.5 [− 2.9–0.6]
**Smoking status**				
Never	2101 (38.5)	717 (39.4)	684 (37.6)	700 (38.5)
Former	2666 (48.9)	843 (44.4)	911 (50.1)	912 (50.2)
Current	688 (12.6)	258 (14.2)	224 (12.3)	206 (11.3)
**Alcohol consumption**				
None	995 (18.2)	344 (18.9)	356 (19.6)	295 (16.2)
Moderate	3181 (58.3)	1070 (58.9)	1042 (57.3)	1069 (58.8)
High	1279 (23.4)	404 (22.2)	421 (23.1)	454 (25.0)
RNFL thickness (μm)	94.8 ± 10.8	106.0 ± 6.3	95.2 ± 2.5	83.2 ± 6.8

Data are presented as mean ± standard deviation, median [interquartile range] or number (%).

CGM, continuous glucose monitoring; HbA1c, hemoglobin A1c; HDL, high-density lipoprotein; NGM, normal glucose metabolism; RNFL, retinal nerve fiber layer; AU, arbitrary units.

*Data shown in the study population with complete data on fasting plasma glucose (n = 5454), 2-h post-load glucose (n = 5180), HbA1c (n = 5449), skin autofluorescence (n = 5132), incremental glucose peak (n = 2407), and CGM-assessed standard deviation (n = 622).

**Data on retinopathy and spherical equivalent were missing for, respectively, n = 137 and n = 275 participants.

### Glucose metabolism status and RNFL thickness

**Figure 2:**
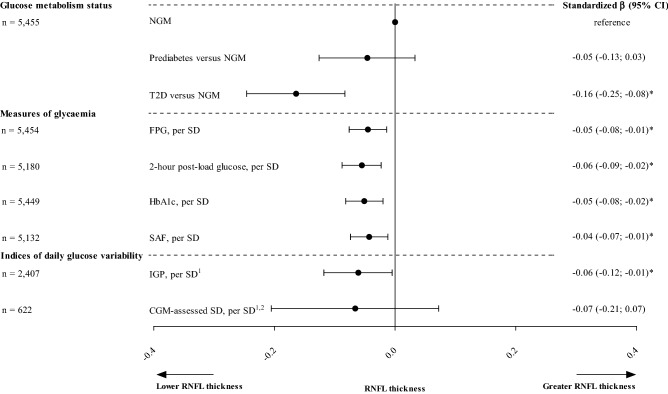
Associations between glucose metabolism status, measures of glycaemia and indices of daily glucose variability with RNFL thickness (per SD). Standardized regression coefficient (stβ) represents the difference in RNFL thickness in SD for type 2 diabetes or prediabetes status versus NGM status, or per SD greater measure of glycaemia or index of daily glucose variability. In the GMS, fasting plasma glucose, 2-h post-load glucose, HbA1c, and SAF study populations, 1 SD corresponds with 10.8 μm for RNFL thickness, 1.5 mmol/L for fasting plasma glucose, 4.0 mmol/L for 2-h post-load glucose, 0.8% or 9.1 mmol/mol for HbA1c, and 0.5 AU for SAF. For incremental glucose peak, 1 SD corresponds with 11.1 μm for RNFL thickness and 2.9 mmol/L for incremental glucose peak. For CGM-assessed standard deviation, 1 SD corresponds with 10.8 μm for RNFL thickness and 0.58 mmol/L for CGM-assessed standard deviation. Variables entered in the models in addition to glucose metabolism status, measures of glycaemia, or indices of daily glucose variability: age, sex, and educational status (low, medium, high), office systolic blood pressure, total cholesterol to HDL cholesterol ratio, use of antihypertensive or lipid-modifying medication (yes/no), waist circumference, smoking status (current, ever, never), and alcohol consumption status (none, low, high). * Indicates statistically significant (*P* < 0.05). ^1^The associations of indices of daily glucose variability with RNFL thickness were additionally adjusted for HbA1c (IGP) or mean sensor glucose (CGM-assessed SD). ^2^Additionally, and only for CGM-assessed SD, we entered ‘visit interval’ in model 1. SD, standard deviation; CI, confidence interval; NGM, normal glucose metabolism; T2D, type 2 diabetes; FPG, fasting plasma glucose; HbA1c, hemoglobin A1c; SAF, skin autofluorescence; IGP, incremental glucose peak; CGM, continuous glucose monitoring.

After full adjustment (model 3), a more adverse GMS was associated with lower RNFL thickness (standardized beta [95%CI], type 2 diabetes versus NGM − 0.16 [− 0.25; − 0.08]; prediabetes versus NGM − 0.05 [− 0.13; 0.03], P for trend = 0.001; Table 2 and Fig. 2).

**Table 2:** Associations of glucose metabolism status, measures of glycaemia, and indices of daily glucose variability with RNFL thickness.

	Number of participants	RNFL, per SD			
		Model 1	Model 2	Model 3	Model 3 + HbA1c/MSG
		stβ (95% CI)	stβ (95% CI)	stβ (95% CI)	stβ (95% CI)
**Glucose metabolism status**					
Prediabetes versus NGM	5455	− 0.07 (− 0.15; 0.003)	− 0.05 (− 0.12; 0.03)	− 0.05 (− 0.13; 0.03)	NA
Type 2 diabetes versus NGM	5455	− **0.19 (**− **0.25;** − **0.12)**	− **0.16 (**− **0.26;** − **0.09)**	− **0.16 (**− **0.25;** − **0.08)**	NA
**Measures of glycaemia**					
Fasting plasma glucose, per SD	5454	− **0.07 (**− **0.09;** − **0.04)**	− **0.05 (**− **0.08;** − **0.02)**	− **0.05 (**− **0.08;** − **0.01)**	NA
2 − hour post − load glucose, per SD	5180	− **0.07 (**− **0.09;** − **0.04)**	− **0.06 (**− **0.09;** − **0.03)**	− **0.06 (**− **0.09;** − **0.02)**	NA
HbA1c, per SD	5449	− **0.06 (**− **0.09;** − **0.03)**	− **0.05 (**− **0.08;** − **0.02)**	− **0.05 (**− **0.08;** − **0.02)**	NA
Skin autofluorescence, per SD	5132	− **0.06 (**− **0.08;** − **0.03)**	− **0.04 (**− **0.04;** − **0.01)**	− **0.04 (**− **0.07;** − **0.01)**	NA
**Indices of daily glucose variability**					
Incremental glucose peak, per SD	2407	− **0.09 (**− **0.13;** − **0.05)**	− **0.07 (**− **0.11;** − **0.03)**	− **0.06 (**− **0.11;** − **0.01)**	− **0.06 (**− **0.12;** − **0.01)**
CGM − assessed standard deviation, per SD	622	− **0.09 (**− **0.18;** − **0.01)**	− **0.09 (**− **0.17;** − **0.001)**	− 0.08 (− 0.17; 0.01)	− 0.07 (− 0.21; 0.07)

Standardized regression coefficient (stβ) represents the difference in RNFL thickness in SD for type 2 diabetes or prediabetes versus NGM or per SD greater measure of glycaemia or index of daily glucose variability. In the GMS, fasting plasma glucose, 2-h post-load glucose, HbA1c, and skin autofluorescence study populations, 1 SD corresponds with 10.8 μm for RNFL thickness, 1.5 mmol/L for fasting plasma glucose, 4.0 mmol/L for 2-h post-load glucose, 0.8% or 9.1 mmol/mol for HbA1c, and 0.5 AU for skin autofluorescence. For incremental glucose peak, 1 SD corresponds with 11.1 μm for RNFL thickness and 2.9 mmol/L for incremental glucose peak. For the CGM-assessed standard deviation, 1 SD corresponds with 10.8 μm for RNFL thickness and 0.58 mmol/L for CGM-assessed standard deviation.

Bold denotes *P* < 0.05.

Variables entered in the models in addition to glucose metabolism status, measures of glycaemia, or indices of daily glucose variability: model 1: none (crude results); model 2: age, sex, and educational status (low, medium, high); model 3: model 2 + office systolic blood pressure, total cholesterol to HDL cholesterol ratio, use of antihypertensive or lipid-modifying medication (yes/no), waist circumference, smoking status (current, ever, never), and alcohol consumption status (none, low, high). In addition, only for incremental glucose peak, model 3 was additionally adjusted for HbA1c (model 3 + HbA1c), and only for CGM-assessed standard deviation, model 3 was additionally adjusted for MSG (model 3 + MSG). Additionally, and only for CGM-assessed SD, we entered ‘visit interval’ in model 1.

AU, arbitrary unit; CI, confidence interval; CGM, continuous glucose monitoring; GMS, glucose metabolism status; HbA1c, hemoglobin A1c; HDL, high-density lipoprotein; NA, not applicable; NGM, normal glucose metabolism; SD, standard deviation; RNFL, retinal nerve fiber layer; MSG, mean sensor glucose.

### Measures of glycaemia and RNFL thickness

After full adjustment (model 3), greater FPG, 2-h post-load glucose, HbA1c, and SAF were statistically significantly associated with lower RNFL thickness (per SD, respectively − 0.05 [− 0.08; − 0.01]; − 0.06 [− 0.09; − 0.02]; − 0.05 [− 0.08; − 0.02]; and − 0.04 [− 0.07; − 0.01]; Table 2 and Fig. 2).

### Indices of daily glucose variability and RNFL thickness

After full adjustment (model 3), greater IGP was statistically significantly associated with lower RNFL thickness (per SD, − 0.06 [− 0.11; − 0.01]; Table 2 and Fig. 2). The association remained statistically significant after additional adjustment for HbA1c (per SD, − 0.06 [− 0.12; − 0.01]). After full adjustment (model 3), CGM-assessed standard deviation was also negatively associated with RNFL thickness, but not statistically significantly (per SD, − 0.08 [− 0.17; 0.01]). The association was similar after further adjustment for mean sensor glucose.

### Interaction analyses

GMS and sex did not modify any of the associations under study. All P-values for interaction are shown in Supplemental Table S4.

### Additional analyses

Quantitatively similar results were observed in a range of sensitivity analyses and are presented in the Supplemental Results section.

## Discussion

The present population-based study had two main findings. First, a more adverse GMS, greater glycaemia (estimated from FPG, 2-h post-load glucose, HbA1c, and SAF) and greater daily glucose variability (estimated from IGP and CGM-assessed standard deviation) were all linearly and –except for CGM-assessed standard deviation– statistically significantly associated with lower RNFL thickness. Second, the associations between indices of daily glucose variability and RNFL thickness did not materially change after additional adjustment for measures of glycaemia.

Our findings are in line with and extend observations from most previous studies^13,19–24^. The present study is the first large population-based study to comprehensively report associations of GMS, measures of glycaemia, and indices of daily glucose variability with RNFL thickness, and also adjust for an extensive set of potential confounders. Additionally, the present study is the first to present associations of SAF, duration of diabetes, and indices of daily glucose variability with RNFL thickness.

Mechanistically, the linearity of the associations of GMS, measures of glycaemia, and indices of daily glucose variability with RNFL thickness may reflect the increasing loss of retinal ganglion cells due to both hyperglycaemia-induced neurotoxicity and impairment of functioning of retinal cells that contribute to metabolic regulation^4,5^. Such impairment of metabolic regulation can predispose retinal ganglion cells to ischemia^5,44^. Importantly, retinal ganglion cells are thought to be highly susceptible to ischemia, since they are highly active and have an energy demand that exceeds that of brain cells^44^.

These findings extend our previous work on the “ticking clock hypothesis”^5,9–12^, which postulates that hyperglycaemia-induced microvascular and neuronal deterioration is a continuous, gradual process that starts in prediabetes, progresses with the onset of type 2 diabetes, and continues during type 2 diabetes^8^. Indeed, we observed that the regression estimate for prediabetes was in between the estimate for type 2 diabetes and the reference category (i.e., NGM), and was directionally and numerically comparable to our previous findings^9–12^. However, the association between prediabetes and RNFL thickness was not statistically significant, which is most likely due to insufficient statistical power. We, therefore, additionally tested for a linear trend with GMS deterioration by using the statistically more powerful P for trend analysis^45^, which was consistent with a linear decrease in RNFL thickness with more adverse GMS. In support, all measures of glycaemia, regardless of whether they reflect shorter (i.e., FPG, 2-h PG, and HbA1c) or longer (i.e., SAF and duration of diabetes) exposure, were consistently linearly associated with RNFL thickness.

Similarly, a likely explanation why the association between CGM-assessed standard deviation and RNFL thickness was not statistically significant is that the statistical power to detect any such association was too low^46^. Indeed, we observed that the association between IGP and RNFL thickness, which included almost fourfold the number of participants (n = 2407 versus n = 622), was statistically significant. Moreover, the strength of the associations of IGP and CGM-assessed standard deviation with RNFL thickness were numerically analogous.

A probable explanation why the association between daily glucose variability and RNFL thickness was not materially altered after additional adjustment for measures of average glycaemia is that daily glucose variability, measures of glycaemia, and GMS represent different underlying constructs^26^. While daily glucose variability reflects oscillating glucose levels, other measures under study reflect exposure to average chronic levels of glycaemia. Mechanistically, substantial glucose fluctuations entail hyperglycaemic peaks, hypoglycaemic nadirs (in individuals with type 2 diabetes treated with agents that can induce hypoglycaemia), or both, which are thought to be potent inducers of retinal ganglion cell apoptosis^26,44^. Whereas hyperglycaemic peaks may be highly neurotoxic, hypoglycaemic nadirs likely hamper retinal ganglion cell metabolism as their key nutrient is glucose^44^.

Our findings can have several implications for clinical practice. First, the strength of the association between type 2 diabetes and RNFL thickness corresponds with 15 years of aging and, thus, indicates that with respect to neurodegeneration substantial “additional aging” occurs in individuals with type 2 diabetes (Supplemental Table S14 shows how this comparison was calculated). Second, RNFL thickness may be a biomarker for the identification of individuals at risk of retinopathy and neuropathy. Use of RNFL thickness measurement is feasible because RNFL thickness assessment is non-invasive^2^, relatively inexpensive^2^ and easier to perform than other tests of early neuronal dysfunction such as 24-h electrocardiogram^9^, magnetic resonance imaging^10,11^, or electromyography^12^. Indeed, RNFL thickness has been found to be a promising early biomarker for other neurodegenerative diseases (e.g., multiple sclerosis)^47^. Third, early glycaemic control, possibly already in prediabetes, is likely crucial in the early prevention of microvascular and neuronal complications^5^. Last, our findings add to growing evidence that control of daily glucose variability besides mean glucose concentrations may be important to prevent microvascular complications^48^.

Strengths of this study are (1) the large size of this population-based cohort with oversampling of individuals with type 2 diabetes, which enabled accurate comparison of individuals with and without diabetes; (2) the extensive number of potential confounders that were considered; (3) the use of state-of-the-art and novel methods (e.g., CGM)^26^ to assess all variables included in this study; and (4) the considerable number of additional analyses, which generally yielded consistent findings.

The study has certain limitations. First, due to the cross-sectional nature of the study, causal inferences should be made with caution^49^. Mechanistically, hyperglycaemia may not only lead to neurodegeneration but the reverse may also be true, thus causing a vicious cycle. Intact neurovascular interaction is required for normal microvascular function and impaired microvascular function may aggravate hyperglycaemia^5,50^. Second, we may have underestimated the strength of the associations of GMS, measures of glycaemia, and daily glucose variability with RNFL thickness if such an association was similar or stronger in participants that were excluded from the study population (who generally tend to be less healthy)^51^. The 2-h post-load glucose and IGP results are most susceptible to this form of selection bias, as no data was available in individuals with the most therapy-intensive diabetes because they were excluded from undergoing an OGTT. Such range restriction may lead to underestimated associations^51^. Third, a single OGTT may misclassify GMS, especially in individuals with prediabetes. Because individuals classified with prediabetes based on their first OGTT are relatively more prone to receive a NGM classification based on their second OGTT^52^, this would likely lead to an underestimation of the association with RNFL thickness in the prediabetes group. Fourth, although we took an extensive set of confounders into account, we cannot fully exclude bias due to unmeasured confounding (e.g., environmental factors such as air pollution)^53^. Fifth, due to the relatively low numbers of participants with data on CGM-based glycaemic indices (n = 622)), and—to a lesser extent—IGP (n = 2407), statistical power of analyses with these determinants was reduced compared to statistical power of analyses with GMS and measures of glycaemia (n = 5132 to n = 5455)^46^. Last, we studied Caucasian individuals aged 40–75 years with access to high-quality diabetes care. Therefore, the generalizability of our results to other populations requires further study.

## Conclusions

In summary, the present population-based study demonstrated that adverse GMS, greater glycaemia, and greater daily glucose variability are associated with lower RNFL thickness, i.e., neurodegeneration, independent of demographics, cardiovascular risk factors, and lifestyle risk factors. Hence, these results suggest that RNFL thickness may be an early biomarker for the identification of individuals at risk of retinopathy and neuropathy. Additionally, the combination of early glycaemic monitoring and early glucose-lowering treatment, possibly already in prediabetes, may contribute to the prevention of RNFL thinning and, ultimately, retinopathy and neuropathy.

## Supplementary Information

Supplementary Information.

## Data Availability

Data are available from The Maastricht Study for any researcher who meets the criteria for access to confidential data; the corresponding author may be contacted to request data.

## Abbreviations

RNFL thickness: Retinal nerve fibre layer thickness
GMS: Glucose metabolism status
OGTT: Oral glucose tolerance test
NGM: Normal glucose metabolism
FPG: Fasting plasma glucose
SAF: Skin autofluorescence
CGM: Continuous glucose monitoring
GV: Glucose variability
IGP: Incremental glucose peak
OCT: Optical coherence tomography
HDL: High-density lipid
BMI: Body-mass index
BP: Blood pressure
eGFR: Estimated glomerular filtration rate
