# Supplementary material for: (Pre)diabetes, glycemia, and daily glucose variability are associated with retinal nerve fiber layer thickness in The Maastricht Study
Source: Sci Rep. 2022 Oct 22;12:17750. doi: 10.1038/s41598-022-22748-2 (PMC9587985; doi:10.1038/s41598-022-22748-2)
Supplement: Supplementary file 1 — Supplementary Information. [file 41598_2022_22748_MOESM1_ESM.docx]

### **Supplemental Material**

Content

Supplemental Methods

Supplemental Results

Supplemental Figures

Supplemental Tables

**Content**

- Supplemental Table S1 Additional general study population characteristics according to tertiles of retinal nerve fiber layer thickness in the study population with complete data on glucose metabolism status
- Supplemental Table S2 General study population characteristics of the included and excluded participants for the study population of glucose metabolism status
- Supplemental Table S3 General study population characteristics of the included and excluded participants for the study populations of indices of daily glucose variability (incremental glucose peak and continuous glucose monitoring-assessed standard deviation)
- Supplemental Table S4 P values of interaction terms with glucose metabolism status and sex
- Supplemental Table S5 Associations of GMS, measures of glycaemia, and indices of daily glucose variability with RNFL thickness after additional adjustment for dietary intake and physical activity (model 4A), for spherical equivalent and intraocular pressure (model 4B), or for eGFR, urinary albumin excretion, history of CVD, plasma biomarkers of low-grade inflammation, retinopathy, and glaucoma (model 4C)
- Supplemental Table S6 Associations of glucose metabolism status, measures of glycaemia, and indices of daily glucose variability with RNFL thickness after exclusion of individuals with retinopathy (model 3A), glaucoma (model 3B), or other types of diabetes (model 3C)
- Supplemental Table S7 Associations or OGTT-based indices of daily glucose variability and CGM-based measures with RNFL thickness
- Supplemental Table S8 Associations of glucose metabolism status, measures of glycaemia, and indices of daily glucose variability with RNFL thickness, where waist circumference was replaced with BMI (model 3A) or educational level was replaced with occupational status (model 3B) or income level (model 3C)
- Supplemental Table S9 Associations of glucose metabolism status, measures of glycaemia, and indices of daily glucose variability with RNFL thickness where office systolic blood pressure was replaced with office diastolic blood pressure (model 3A), 24-hour ambulatory systolic blood pressure (model 3B), or 24-hour ambulatory diastolic blood pressure (model 3C)
- Supplemental Table S10 Standardized regression coefficients of skin autofluorescence with retinal nerve fiber thickness after additional adjustment for fasting plasma glucose, 2-hour post-load glucose, or HbA1c
- Supplemental Table S11 Standardized regression coefficients of incremental glucose peak and CGM-assessed standard deviation with retinal nerve fiber thickness after replacement of HbA1c with fasting plasma glucose or skin autofluorescence, or mean sensor glucose with fasting plasma glucose, skin autofluorescence, or HbA1c
- Supplemental Table S12 Associations of continuous glucose monitoring-assessed standard deviation with retinal nerve fiber thickness after exclusion of individuals with less than 2 days of CGM data available (model 3A), individuals with CGM data gaps (model 3B), or with a ‘visit interval’ (Model 3C)
- Supplemental Table S13 Associations of CGM-assessed standard deviation and mean sensor glucose with retinal nerve fiber thickness estimated with ridge regression and presented for different degrees of penalization
- Supplemental S14 Associations of duration of diabetes and age with retinal nerve fiber layer thickness

**Supplemental Methods**

*Performance of optical coherence tomography (OCT) scans*

For a subset of participants (n=227) OCT scans were performed as part of a catch up visit. For these participants only there was a median time interval of 4.4 (interquartile range 4.0-5.9) years between OCT and all other measurements. We checked whether this affected the associations and this was not the case (data not shown).

*Grading of OCT circle scans*

OCT scans were considered of sufficient quality if all the following criteria were met: good centering of the circular scan on the optic nerve head (examples of good, poor and very poor centering are shown in Supplemental Figure S1); complete (data of all 768 voxels was available); automatic quality ≥15 dB (an example of a scan with poor quality imaging is shown in Supplemental Figure S2); no measurement error present (examples of all assessed measurement errors are shown in Supplemental Figure S2). The percentage of agreement for selection of scans with sufficient quality ranged between 90% and 94% for four trained graders and was 70% for one grader (n=50 OCT scans per comparison).

*Assessment of advanced glycation endproducts*

Advanced glycation endproducts (AGEs) were assessed with the AGE Reader (DiagnOptics Technologies BV, Groningen, the Netherlands). In brief, the AGE Reader is a desktop device that uses the characteristic fluorescent properties of certain AGEs to quantify their accumulation in the skin as skin autofluorescence (SAF; arbitrary units [AU]).^1^ The AGE Reader illuminates a skin surface of 4 cm^2^, shielded from other light, and uses the ratio of the reflection of fluorescent light (wavelength 420 to 600 nm) to non-fluorescent light (300–420 nm) to calculate SAF.

Due to the use of different versions of calibration software absolute SAF values assessed before the 4^th^ September 2012 were 0.5 arbitrary units higher than the SAF values assessed after this date. To realign absolute SAF values we recalculated the SAF values assessed before the 4^th^ of September 2012 by subtracting 0.5 arbitrary units from the SAF values assessed before the 4^th^ September 2012.

*Assessment of OGTT-based and CGM-based glycemic indices*

Incremental glucose peak (IGP), maximum glucose peak (i.e., the highest of the seven oral glucose tolerance test [OGTT] time points) and 1-h post-load glucose value were assessed in venous OGTT-derived samples. We have recently shown that the 1-hour post-load glucose value, maximum glucose peak, and IGP correlated most strongly with continuous glucose monitoring (CGM)-based indices of daily glucose variability^2^. Of these indices, the association between IGP and CGM-measured daily glucose variability indices was most consistent across different GMS strata, and, hence, IGP is considered the preferred OGTT-based index of daily glucose variability.

The rationale and methodology of CGM (iPro2 and Enlite Glucose Sensor; Medtronic, Tolochenaz, Switzerland) in The Maastricht Study have been described previously.^2^ From 19 September 2016 until 13 September 2018, all new participants were invited to undergo CGM as part of their regular measurements at The Maastricht Study. To accelerate the inclusion process and to ensure the inclusion of a sufficient number of participants with prediabetes and type 2 diabetes, a selected group of participants who had recently visited The Maastricht Study was re-invited to undergo CGM as a separate research visit (‘catch-up visit’). For individuals who participated in the catch-up visit, there was an average time period (“visit interval”) of 2.1 years between CGM and other measurements. The CGM device was worn on the lower abdomen and recorded subcutaneous interstitial glucose values (range: 2.2 - 22.2 mmol/L) every five minutes for a 7-day period. Participants were asked to perform self-measurements of blood glucose four times daily (Contour Next; Ascensia Diabetes Care, Mijdrecht, the Netherlands) for retrospective calibration of the CGM device. Participants were blinded to the CGM recording, but not to the self-measured values. Diabetes medication use was allowed during the CGM period, and no dietary or physical activity instructions were given.

The first 24 hours of CGM were excluded, because of insufficient calibration. Next, we excluded individuals of whom less than 24 hours of recording (less than one data day) remained. Then, we calculated mean sensor glucose (mmol/L), standard deviation (mmol/L), coefficient of variation (standard deviation / mean sensor glucose * 100%), and time in range (TIR; i.e., % of time between 3.9 – 10.0 mmol/L) for the total recording period. Based on international consensus, we used standard deviation and coefficient of variation as indices of daily glucose variability.^3^ The CGM-assessed coefficient of variation, which is intrinsically adjusted for mean sensor glucose, was not used as main determinant, as use of a ratio variable may introduce bias.^4, 5^ TIR is an emerging glycemic index that is partly determined by daily glucose variability.^6^

*Assessment of retinopathy*

All signs of retinopathy were graded from fundus photos by an ophthalmologist according the Early Treatment Diabetic Retinopathy Study Research Group (ETDRS) criteria.^7^ Presence of retinopathy was defined according to the American Academy of ophthalmology preferred practice guidelines.^8^

*Additional statistical analysis*

We performed a range of additional analyses. First, we repeated the analyses with additional adjustment for lifestyle factors (dietary intake, physical activity) or ocular variables (spherical equivalent, intraocular pressure).^9-11^ Adjustment for these potential confounders was not included in the main analyses because data were missing for a relatively large number of participants (up to n=768 had missing data on one or more of these variables). Second, we additionally adjusted for kidney variables (eGFR and urinary albumin excretion), history of cardiovascular disease, plasma biomarkers of low-grade inflammation, retinopathy, and glaucoma. We adjusted for these covariates in a separate model because they may be confounders but may also (in part) be mediators or descendants of the outcome.^12^ Third, we performed analyses in which participants with retinopathy, glaucoma, or other types of diabetes were excluded. Fourth, we replaced IGP with other OGTT-based indices of daily glucose variability (i.e., maximum glucose peak and 1-hour post-load glucose). We did not include maximum glucose peak and 1-hour post-load glucose in the main analyses because they are known to correlate less strongly than IGP with CGM-assessed indices of daily glucose variability in GMS-stratified groups.^2^ Fifth, we replaced CGM-assessed standard deviation with other CGM-assessed measures of daily glucose variability. Sixth, we studied the association between mean sensor glucose and retinal nerve fibre layer thickness. Although the sample size of the CGM study population was relatively small (n=622), CGM-based glycaemic mean sensor glucose may be less susceptible to measurement error than other measures of glycaemia under study, as mean sensor glucose constitutes the average of a large number of glucose concentrations.^2, 13^ Seventh, we replaced waist circumference with BMI; educational status with occupational status or income level; and office systolic BP with office diastolic BP, systolic or diastolic 24-hour ambulatory BP. Eighth, and only for SAF, we additionally adjusted the association between SAF and RNFL thickness for FPG, 2-hour post-load glucose, or HbA1c. We additionally adjusted for these measures to investigate whether the association between SAF and RNFL thickness was independent of short to middle long-term exposure to hyperglycaemia. Ninth, and only for IGP and CGM-assessed standard deviation, replaced we respectively HbA1c with FPG or SAF, and mean sensor glucose with FPG, SAF, or HbA1c. Tenth, and only for CGM-assessed standard deviation, repeated we the analyses after exclusion of individuals with insufficient CGM recording days, with CGM recording data gaps, or with a visit interval. Eleventh, because of the strong correlation between CGM-assessed standard deviation and mean sensor glucose,^14^ we repeated the main analysis using ridge regression, which is a valid method to counter potential instability caused by multicollinearity (additional information is provided below).^15^ Last, we studied the association between duration of diabetes and RNFL thickness. Data on duration of diabetes were only available for individuals with type 2 diabetes (n=982).

*Ridge regression*

Because we presumed the reliability of ‘model 3 + mean sensor glucose’ to be negatively impacted by multicollinearity, due to the strong correlation between CGM-assessed standard deviation and mean sensor glucose (rho = 0.69),^15^ we additionally performed ridge regression. It is a L2-regularized form of linear regression and a valid statistical method to counter a degree of model instability caused by multicollinearity.^16^ Ridge regression estimates are computed according to the combination of the residual sum of squares characteristic of regular linear regression and predefined penalization of the coefficients (i.e., $Ridge= RSS+ \frac{1}{n}* \lambda* \sum_{j=1}^{p} \beta_{j}^{2}$, where RSS is the residual sum of squares, n is the sample size, λ is the chosen amount of penalization, and $\sum_{j=1}^{p} \beta_{j}^{2}$ represents the sum of all squared regression coefficients). As such, it slightly biases the regression coefficients and can strongly reduce inflated variances that arise when high multicollinearity is present. We pragmatically chose the level of penalization based on the lambda (λ) required to reduce the variance inflation factor (VIF) of model 3.1 back to the VIF of model 3 (or halfway back). The ridge regression results are presented as: standardized regression coefficient (st.β) (95%CI), P value. The median st.βs (95%CIs) were estimated with use of resampling (1,000 bootstraps).

**Supplemental Results**

Additional analyses

Quantitatively similar results were observed in a range of sensitivity analyses. First, associations remained similar after we additionally adjusted for physical activity and dietary habits, or spherical equivalent and intraocular pressure (Supplemental Table S5). Second, associations were generally comparable when we additionally adjusted for kidney variables, history of cardiovascular disease, plasma biomarkers of low-grade inflammation, retinopathy, and glaucoma (Supplemental Table S5). Third, associations were similar when we excluded participants with retinopathy, glaucoma, or other types of diabetes (Supplemental Table S6). Fourth, associations were numerically comparable when we replaced IGP with other OGTT-based indices of glucose variability and CGM-assessed standard deviation with other CGM-assessed measures that reflect aspects of daily glucose variability, including coefficient of variation (CV), time-in-range (TIR), time below range (TBR); and time above range (TAR; Supplemental Table S7). Fifth, the strength of the association of CGM-assessed mean sensor glucose with RNFL thickness was numerically comparable to the strength of the associations of measures of glycaemia with RNFL thickness (Supplemental Table S7). Sixth, associations remained similar after replacement of waist circumference with BMI; of educational status with occupational status or income level; and of office systolic BP with office diastolic BP, or systolic or diastolic 24-hour ambulatory BP (Supplemental Tables S8 and S9). Seventh, the association between SAF was not altered after additional adjustment for FPG, 2-hour post-load glucose, or HbA1c (Supplemental Table S10). Eighth, associations of IGP and CGM-assessed standard deviation with RNFL thickness were similar after replacement of HbA1c or mean sensor glucose with other measures of glycaemia (Supplemental Table S11). Ninth, the associations between CGM-assessed standard deviation and RNFL thickness were similar after exclusion of individuals with insufficient recording days or recording data gaps (Supplemental Table S12). Exclusion of participants with a visit interval, most of whom had type 2 diabetes,^2^ strongly attenuated the association between CGM-assessed standard deviation and RNFL thickness. Tenth, ridge regression did not yield materially different results for the association of CGM-assessed standard deviation with RNFL thickness (Supplemental Table S13). Last, after full adjustment (model 3), longer duration of diabetes was significantly associated with lower RNFL thickness (per SD, -0.07 [-0.13; -0.001]; Supplemental Table S14).

**References**

1. van Eupen MG, Schram MT, van Sloten TT, Scheijen J, Sep SJ, van der Kallen CJ, Dagnelie PC, Koster A, Schaper N, Henry RM, Kroon AA, Smit AJ, Stehouwer CD and Schalkwijk CG. Skin Autofluorescence and Pentosidine Are Associated With Aortic Stiffening: The Maastricht Study. *Hypertension*. 2016;68:956-63.

2. Foreman YD, Brouwers MCGJ, van der Kallen CJH, Pagen DME, van Greevenbroek MMJ, Henry RMA, Koster A, Wesselius A, Schaper NC and Stehouwer CDA. Glucose Variability Assessed with Continuous Glucose Monitoring: Reliability, Reference Values, and Correlations with Established Glycemic Indices-The Maastricht Study. *Diabetes Technol Ther*. 2020;22:395-403.

3. Danne T, Nimri R, Battelino T, Bergenstal RM, Close KL, DeVries JH, Garg S, Heinemann L, Hirsch I, Amiel SA, Beck R, Bosi E, Buckingham B, Cobelli C, Dassau E, Doyle FJ, 3rd, Heller S, Hovorka R, Jia W, Jones T, Kordonouri O, Kovatchev B, Kowalski A, Laffel L, Maahs D, Murphy HR, Norgaard K, Parkin CG, Renard E, Saboo B, Scharf M, Tamborlane WV, Weinzimer SA and Phillip M. International Consensus on Use of Continuous Glucose Monitoring. *Diabetes Care*. 2017;40:1631-1640.

4. Curran-Everett D. Explorations in statistics: the analysis of ratios and normalized data. *Adv Physiol Educ*. 2013;37:213-9.

5. Rodbard D. Glycemic variability: challenges in interpretation. *Diabetes Technol Ther*. 2015;17:370-2.

6. Rodbard D. Glucose Time In Range, Time Above Range, and Time Below Range Depend on Mean or Median Glucose or HbA1c, Glucose Coefficient of Variation, and Shape of the Glucose Distribution. *Diabetes Technol Ther*. 2020.

7. Grading diabetic retinopathy from stereoscopic color fundus photographs--an extension of the modified Airlie House classification. ETDRS report number 10. Early Treatment Diabetic Retinopathy Study Research Group. *Ophthalmology*. 1991;98:786-806.

8. Committee AAoOPPPRV. Diabetic Retinopathy PPP 2019. 2019;2020.

9. Bikbov MM, Kazakbaeva GM, Gilmanshin TR, Zainullin RM, Arslangareeva, II, Salavatova VF, Bikbova GM, Panda-Jonas S, Nikitin NA, Zaynetdinov AF, Nuriev IF, Khikmatullin RI, Uzianbaeva YV, Yakupova DF, Aminev SK and Jonas JB. Axial length and its associations in a Russian population: The Ural Eye and Medical Study. *PLoS One*. 2019;14:e0211186.

10. Haeger A, Costa AS, Schulz JB and Reetz K. Cerebral changes improved by physical activity during cognitive decline: A systematic review on MRI studies. *Neuroimage Clin*. 2019;23:101933.

11. Poulose SM, Miller MG, Scott T and Shukitt-Hale B. Nutritional Factors Affecting Adult Neurogenesis and Cognitive Function. *Adv Nutr*. 2017;8:804-811.

12. Schisterman EF, Cole SR and Platt RW. Overadjustment bias and unnecessary adjustment in epidemiologic studies. *Epidemiology*. 2009;20:488-95.

13. Battelino T, Danne T, Bergenstal RM, Amiel SA, Beck R, Biester T, Bosi E, Buckingham BA, Cefalu WT, Close KL, Cobelli C, Dassau E, DeVries JH, Donaghue KC, Dovc K, Doyle FJ, 3rd, Garg S, Grunberger G, Heller S, Heinemann L, Hirsch IB, Hovorka R, Jia W, Kordonouri O, Kovatchev B, Kowalski A, Laffel L, Levine B, Mayorov A, Mathieu C, Murphy HR, Nimri R, Norgaard K, Parkin CG, Renard E, Rodbard D, Saboo B, Schatz D, Stoner K, Urakami T, Weinzimer SA and Phillip M. Clinical Targets for Continuous Glucose Monitoring Data Interpretation: Recommendations From the International Consensus on Time in Range. *Diabetes Care*. 2019;42:1593-1603.

14. Rodbard D. The challenges of measuring glycemic variability. *J Diabetes Sci Technol*. 2012;6:712-5.

15. Vatcheva KP, Lee M, McCormick JB and Rahbar MH. Multicollinearity in Regression Analyses Conducted in Epidemiologic Studies. *Epidemiology (Sunnyvale)*. 2016;6.

16. Dormann CF, Elith J, Bacher S, Buchmann C, Carl G, Carre G, Marquez JRG, Gruber B, Lafourcade B, Leitao PJ, Munkemuller T, McClean C, Osborne PE, Reineking B, Schroder B, Skidmore AK, Zurell D and Lautenbach S. Collinearity: a review of methods to deal with it and a simulation study evaluating their performance. *Ecography*. 2013;36:27-46.

**Supplemental Figures**

S1a
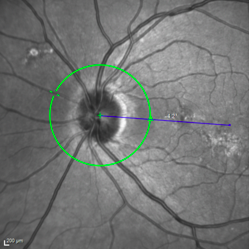
 S1b
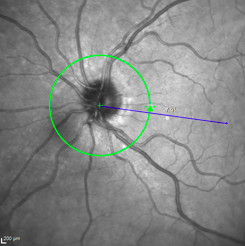


S1c
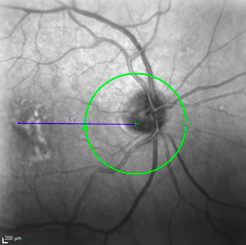


Supplemental Figure S1 Examples of quality of centering of circular scans on the optic nerve head

Supplemental Figure S1 shows examples of quality of centering of the circular scan on the optic nerve head: S1a shows good quality, S1b shows poor quality, and S1c shows very poor quality.

**
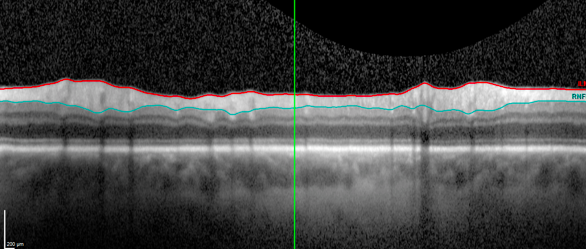
**

S2a

S2b
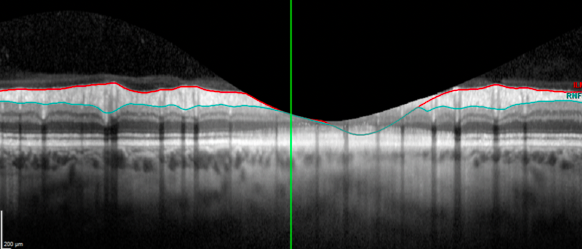


S2c
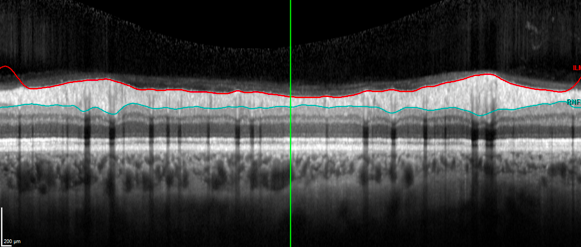


S2d
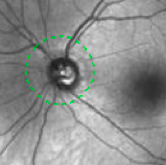


S2e
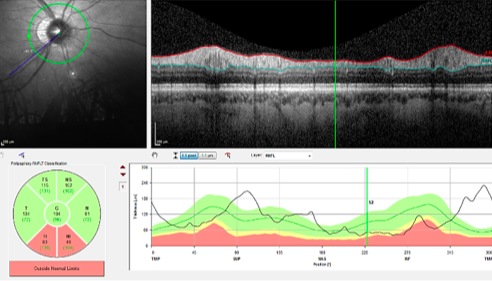


S2f
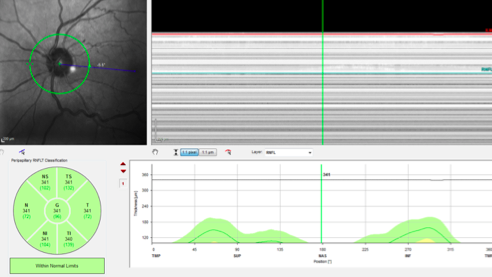


S2g
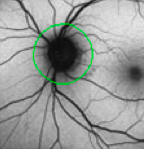


Supplemental Figure S2 Examples of poor quality and scan errors

S2a: Example of poor imaging quality (Signal-to-noise ratio<15 dB); S2b: OCT device too close to the eye; S2c: RNFL layer incorrectly defined; S2d: incorrect circle position (dashed line); S2e: participant does not look in the correct direction; S2f: technical problem with OCT device; S2g: autofluorescence on.

Abbreviations: OCT, optical coherence tomography; RNFL, retinal nerve fiber layer thickness.

**Supplemental Tables**

Supplemental Table S1 Additional general study population characteristics according to tertiles of retinal nerve fiber layer thickness in the study population with complete data on glucose metabolism status

|  |  | **RNFL thickness** |  |  |  |
| --- | --- | --- | --- | --- | --- |
| **Characteristic** | Total study  population  (n = 5,455) | Tertile 1 (high)  (n =1,818) | Tertile 2 (middle)  (n =1,819) | Tertile 3 (low)  (n =1,818) | Number of participants with missing data |
| BMI (kg/m2) | 26.8 ± 4.4 | 26.8 ± 4.5 | 26.7 ± 4.3 | 26.9 ± 4.3 | 1 |
| Income level (euro) | 1,856 [1,502-2,386] | 1,856 [1,502-2,386] | 1,875 [1,503-2,386] | 1,856 [1,503-2,386] | 1,288 |
| Occupational status |  |  |  |  | 3,618 |
| Low | 572 (31.1) | 195 (34.2) | 190 (31.2) | 187 (28.4) |  |
| Intermediate | 666 (36.3) | 207 (36.3) | 231 (37.9) | 228 (34.7) |  |
| High | 599 (32.6) | 168 (29.5) | 188 (30.9) | 243 (36.9) |  |
| Systolic ambulatory 24-hour blood pressure (mmHg) | 118.5 ± 11.3 | 117.8 ± 11.6 | 118.6 ± 11.2 | 119.3 ± 11.3 | 535 |
| Diastolic ambulatory 24-hour blood pressure (mmHg) | 72.9 ± 7.1 | 72.4 ± 7.2 | 73.0 ± 7.1 | 73.3 ± 6.9 | 535 |
| Dutch Healthy diet score (points) | 84.0 ± 15.0 | 84.5 ± 15.1 | 84.6 ± 15.1 | 82.8 ± 14.9 | 388 |
| Physical activity (hours/day) | 2.0 ± 0.7 | 2.0 ± 0.7 | 2.0 ± 0.7 | 2.0 ± 0.7 | 768 |
| Spherical equivalent (diopter) | 0.13 [-1.19-1.06] | 0.6 [-0.4-1.6] | 0.1 [-1.1-1.1] | -0.5 [-2.9-0.6] | 275 |
| Intraocular pressure (mmHg) | 14.0 [11.7-16.0] | 13.5 [11.5-15.7] | 13.8 [11.5-15.7] | 14.3 [12.0-16.5] | 319 |
| Glaucoma | 234 (4.6) | 63 (3.7) | 53 (3.1) | 118 (6.9) | 319 |
|  |  |  |  |  |  |
| History of CVD | 1,337 (16.9) | 279 (15.4) | 293 (16.2) | 324(17.9) | 101 |
| eGFR, ml/min/1.73m2 | 82.3 ± 14.0 | 83.0 ± 13.8 | 82.1 ± 13.9 | 81.9 ± 14.1 | 3 |
| Urinary albumin excretion (mg/24 hours) | 5.4 [3.4-10.0] | 5.2 [3.4-9.8] | 5.4 [3.4-9.5] | 5.5 [3.4-10.6] | 23 |
| Albuminuria | 424 (7.8) | 130 (7.2) | 137 (7.6) | 157 (8.7) | 23 |
| Biomarkers of low-grade inflammation |  |  |  |  | 3,049 |
| C-reactive protein, µg/ml  Serum amyloid A, µg/ml  Interleukin-6, pg/ml  Interleukin-8, pg/ml  Tumor necrosis factor α, pg/ml | 1.2 [0.6-2.7]  3.2 [2.0-5.4]  0.6 [0.4-0.9]  4.0 [3.2-5.2]  2.2 [1.9-2.6] | 1.2 [0.6-2.7]  3.2 [2.0-5.5]  0.6 [0.4-0.9]  3.9 [3.2-4.9]  2.1 [1.8-2.6] | 1.2 [0.6-2.6]  3.2 [2.1-5.2]  0.5 [0.4-0.8]  4.1 [3.2-5.2]  2.2 [1.9-2.5] | 1.2 [0.7-2.8]  3.3 [2.1-5.6]  0.6 [0.4-1.0]  4.2 [3.2-5.3]  2.2 [1.9-2.6] |  |
| Duration of diabetes (years) | 4.0 [0-9.0] | 3.0 [0-9.0] | 3.0 [0-8.0] | 4.0 [1.0-10.8] | 257 |
| Maximum glucose peak (mmol/L) | 9.5 [7.9-12.4] | 9.3 [7.8-11.7] | 9.5 [7.9-12.2] | 9.8 [8.1-13.4] | 3,048 |
| 1-hour post-load glucose (mmol/L) | 8.6 [6.5-11.9] | 8.4 [6.3-11.2] | 8.6 [6.6-11.7] | 8.8 [6.7-12.6] | 2,924 |
| CGM: indices and methodology |  |  |  |  | 4,833 |
| Mean sensor glucose, mmol/L | 6.1 [5.9-6.8] | 6.1 [5.7-6.6] | 6.0 [5.7-6.7] | 6.1 [5.7-6.9] |  |
| Coefficient of variation, % | 14.5 [11.7-17.8] | 14.6 [11.6-17.4] | 14.1 [11.5-17.6] | 14.8 [11.9-18.7] |  |
| Time in range, % | 99.7 [96.8-100.0] | 99.7 [96.8-100.0] | 99.7 [97.8-100.0] | 99.7 [96.1-100.0] |  |
| CGM during regular visit | 409 (65.8) | 123 (63.4) | 135 (65.9) | 151 (67.7) |  |
| CGM during ‘catch-up visit’ | 213 (34.2) | 71 (36.6) | 70 (34.1) | 72 (32.2) |  |
| Insufficient recording day, yes vs. no | 8 (1.3) | 1 (0.5) | 2 (1.0) | 5 (2.2) |  |
| CGM recording data gap, yes vs. no | 49 (7.9) | 18 (9.3) | 14 (6.8) | 17 (7.6) |  |
| CGM visit interval* (years) | 2.1 [2.0-2.2] | 2.1 [2.0-2.2] | 2.1 [2.1-2.2] | 2.1 [2.0-2.2] |  |

Data are presented as mean ± standard deviation, median [interquartile range] or number (%).

Abbreviations: BMI, body-mass index; CGM, continuous glucose monitoring, CVD, cardiovascular disease; eGFR, estimated glomerular filtration rate.

* Of ‘catch-up visit’ participants only (n=213)

Supplemental Table S2 General study population characteristics of the included and excluded participants for the study population of glucose metabolism status

|  | Included study  population  (n = 5,455) | Missing data in/excluded | Excluded study population  (n =2,550) |
| --- | --- | --- | --- |
| **Characteristic** |  |  |  |
| Age (years) | 59.5 ± 8.6 | 0/0 | 60.6 ± 8.7 |
| Men | 2,665 (48.9) | 0/0 | 1356 (53.2) |
| Educational level |  | 0/116 |  |
| Low | 1,914 (35.1) |  | 821 (33.7) |
| Medium | 1,519 (27.8) |  | 647 (26.6) |
| High | 2,022 (37.1) |  | 966 (39.7) |
| Glucose metabolism status |  | 0/0 |  |
| NGM | 3,366 (61.7) |  | 1,479 (58.0) |
| Prediabetes | 820 (15.0) |  | 370 (14.5) |
| Type 2 diabetes | 1,239 (22.7) |  | 681 (26.7) |
| Other type of diabetes | 30 (0.5) |  | 20 (0.8) |
| Measures of glycaemia |  |  |  |
| Fasting plasma glucose (mmol/L) | 5.9 ± 1.5 | 1/0 | 6.0 ± 1.8 |
| 2-hour post-load glucose (mmol/L) | 6.2 [4.9-8.6] | 275/212 | 6.2 [4.9-8.8] |
| HbA1c (mmol/mol) | 39.2 ± 9.1 | 6/8 | 40.6 ± 10.4 |
| HbA1c (%) | 5.7 ± 0.8 | 6/8 | 5.9 ± 1.0 |
| SAF (AU) | 2.2 ± 0.5 | 323/296 | 2.2 ± 0.5 |
| Indices of daily glucose variability |  |  |  |
| Incremental glucose peak (mmol/L) | 4.1 [2.7-6.5] | 3048/1368 | 4.2 [2.7-6.6] |
| CGM-assessed standard deviation (mmol/L) | 0.86 [0.68-1.21] | 4833/2319 | 0.79 [0.68-1.08] |
| Use of glucose-modifying medication, yes vs. no | 927 (17.0) | 0/6 | 536 (21.1) |
| Duration of diabetes (years) | 4.0 [0-9.0] | 257/155 | 4.5 [1.0-10.0] |
| Waist circumference, men (cm) | 100.4 ± 11.6 | 0/2 | 102.1 ± 12.5 |
| Waist circumference, women (cm) | 89.1 ± 12.5 | 0/3 | 90.7 ± 13.7 |
| Total-to-HDL cholesterol ratio | 3.4 [2.8-4.2] | 0/4 | 3.5 [2.8-4.4] |
| Use of lipid-modifying medication, yes vs. no | 1,687 (30.9) | 0/6 | 864 (34.0) |
| Office systolic blood pressure (mmHg) | 133.2 ± 17.7 | 0/3 | 134.6 ± 18.3 |
| Office diastolic blood pressure (mmHg) | 75.5 ± 9.8 | 1/3 | 75.4 ± 9.8 |
| Use of antihypertensive medication, yes vs. no | 1,983 (36.4) | 0/6 | 1039 (40.8) |
| Smoking status |  | 0/65 |  |
| Never | 2,101 (38.5) |  | 858 (34.5) |
| Former | 2,666 (48.9) |  | 1253 (50.4) |
| Current | 688 (12.6) |  | 374 (15.1) |
| Alcohol consumption |  | 0/66 |  |
| None | 995 (18.2) |  | 478 (19.2) |
| Moderate | 3,181 (58.3) |  | 1,447 (58.3) |
| High | 1,279 (23.4) |  | 559 (22.5) |
| RNFL thickness (μm) | 94.8 ± 10.8 | 0/2454* | 95.1 ± 11.5 |

Data are presented as mean ± standard deviation, median [interquartile range] or number (%).

* Number of participants with missing data represents the number of participants that missed RNFL thickness assessment in both eyes or had a RNFL thickness assessment of insufficient quality for both eyes.

Abbreviations: CGM, continues glucose monitoring; HbA1c, hemoglobin A1c; HDL, high-density lipoprotein; NGM, normal glucose metabolism; RNFL, retinal nerve fiber layer; SAF, skin autofluorescence AU, arbitrary units; NA, not applicable.

Supplemental Table S3 General study population characteristics of the included and excluded participants for the study populations of indices of daily glucose variability (incremental glucose peak and continuous glucose monitoring-assessed standard deviation)

|  | Incremental glucose peak | | | Continuous glucose monitoring-assessed standard deviation | | |
| --- | --- | --- | --- | --- | --- | --- |
|  | Included study  population  (n = 2,407) | Missing data in/excluded | Excluded study population  (n =5,598) | Included study  population  (n = 622) | Missing data in/excluded | Excluded study population   (n =7383) |
| **Characteristic** |  |  |  |  |  |  |
| Age (years) | 59.5 ± 8.2 | 0/0 | 60.0 ± 8.9 | 59.8 ± 8.5 | 0/0 | 59.8 ± 8.7 |
| Men | 1,211 (50.3) | 0/0 | 2810 (50.2) | 318 (51.1) | 0/0 | 3703 (50.2) |
| Educational level |  | 0/116 |  |  | 0/116 |  |
| Low | 794 (33.0) |  | 1941 (35.4) | 205 (33.0) |  | 2530 (34.8) |
| Medium | 691 (28.7) |  | 1475 (26.9) | 168 (27.0) |  | 1998 (27.5) |
| High | 922 (38.3) |  | 2066 (37.7) | 249 (40.0) |  | 2739 (37.7) |
| Glucose metabolism status |  | 0/0 |  |  | 0/0 |  |
| NGM | 1,455 (60.4) |  | 3,390 (60.6) | 328 (52.7) |  | 4517 (61.2) |
| Prediabetes | 430 (17.9) |  | 760 (13.6) | 140 (22.5) |  | 1050 (14.2) |
| Type 2 diabetes | 522 (21.7) |  | 1398 (25.0) | 152 (24.4) |  | 1768 (23.9) |
| Other type of diabetes | 0 (0.0) |  | 50 (0.9) | 2 (0.3) |  | 48 (0.7) |
| Measures of glycaemia |  |  |  |  |  |  |
| Fasting plasma glucose (mmol/L) | 5.8 ± 1.1 | 0/1 | 6.0 ± 1.8 | 5.9 ± 1.5 | 0/1 | 5.9 ± 1.6 |
| 2-hour post-load glucose (mmol/L) | 6.4 [5.1-9.1] | 0/487 | 6.1 [4.9-8.4] | 6.8 [5.3-9.4] | 29/458 | 6.1 [4.9-8.6] |
| HbA1c (mmol/mol) | 38.9 ± 6.9 | 0/14 | 40.0 ± 10.5 | 39.6 ± 8.8 | 0/14 | 39.7 ± 9.6 |
| HbA1c (%) | 5.7 ± 0.6 | 0/14 | 5.8 ± 1.0 | 5.8 ± 0.8 | 0/14 | 5.8 ± 0.9 |
| SAF (AU) | 2.2 ± 0.4 | 112/507 | 2.2 ± 0.5 | 2.1 ± 0.5 | 49/570 | 2.2 ± 0.5 |
| Indices of daily glucose variability |  |  |  |  |  |  |
| Incremental glucose peak (mmol/L) | 4.1 [2.7-6.5] | 0/4416 | 4.2 [2.7-6.6] | 4.4 [3.1-6.9] | 75/4341 | 4.1 [2.7-6.4] |
| CGM-assessed standard deviation (mmol/L) | 0.84 [0.68-1.16] | 1860/5292 | 0.84 [0.69-1.23] | 0.86 [0.68-1.21] | 0/7152 | 0.79 [0.68-1.08] |
| Use of glucose-modifying medication, yes vs. no | 350 (14.5) | 0/6 | 1113 (19.9) | 88 (14.1) | 0/6 | 1375 (18.6) |
| Duration of diabetes | 2.0 [0.0-6.0] | 97/315 | 5.0 [1.0-11.0] | 1.0 [0.0-4.0] | 22/390 | 4.0 [1.0-10.0] |
| Waist circumference, men (cm) | 100.7 ± 11.3 | 0/2 | 101.1 ± 12.2 | 102.6 ± 11.8 | 0/2 | 100.8 ± 12.0 |
| Waist circumference, women (cm) | 89.4 ± 12.2 | 0/3 | 89.6 ± 13.2 | 90.8 ± 12.4 | 0/3 | 89.4 ± 12.9 |
| Total-to-HDL cholesterol ratio | 3.4 [2.8-4.2] | 0/4 | 3.4 [2.8-4.3] | 3.5 [2.8-4.4] | 0/4 | 3.4 [2.8-4.2] |
| Use of lipid-modifying medication, yes vs. no | 735 (30.5) | 0/6 | 1816 (32.5) | 159 (25.6) | 0/6 | 2392 (32.4) |
| Office systolic blood pressure (mmHg) | 134.0 ± 17.8 | 0/3 | 133.5 ± 18.0 | 134.1 ± 18.3 | 0/3 | 133.6 ± 17.9 |
| Office diastolic blood pressure (mmHg) | 76.3 ± 10.0 | 0/4 | 75.2 ± 9.7 | 75.7 ± 10.2 | 0/4 | 75.5 ± 9.8 |
| Use of antihypertensive medication, yes vs. no | 878 (36.5) | 0/6 | 2144 (38.3) | 231 (37.1) | 0/6 | 2791 (37.8) |
| Smoking status |  | 0/65 |  |  | 0/65 |  |
| Never | 863 (35.9) |  | 2096 (37.9) | 238 (38.3) |  | 2721 (37.2) |
| Former | 1,238 (51.4) |  | 2681 (48.5) | 308 (49.5) |  | 3611 (49.3) |
| Current | 306 (12.7) |  | 756 (13.7) | 76 (12.2) |  | 986 (13.5) |
| Alcohol consumption |  | 0/66 |  |  | 0/66 |  |
| None | 401 (16.7) |  | 1072 (19.4) | 104 (16.7) |  | 1369 (18.7) |
| Moderate | 399 (58.1) |  | 3,229 (58.4) | 400 (64.3) |  | 4228 (57.8) |
| High | 607 (25.2) |  | 1231 (22.3) | 118 (19.0) |  | 1720 (23.5) |
| RNFL thickness (μm) | 94.5 ± 11.1 | 0/2454* | 95.1 ± 10.6 | 94.5 ± 10.8 | 0/2454* | 94.9 ± 10.9 |

Data are presented as mean ± standard deviation, median [interquartile range] or number (%).

* Number of participants with missing data represents the number of participants that missed RNFL thickness assessment in both eyes or had a RNFL thickness assessment of insufficient quality for both eyes.

Abbreviations: CGM, continues glucose monitoring; HbA1c, hemoglobin A1c; HDL, high-density lipoprotein; NGM, normal glucose metabolism; RNFL, retinal nerve fiber layer; SAF, skin autofluorescence AU, arbitrary units; NA, not applicable.

Supplemental Table S4 P values of interaction terms with glucose metabolism status and sex

|  |  | **Prediabetes** | **Type 2 diabetes** | **Sex** |
| --- | --- | --- | --- | --- |
|  | Number of participants | P-value | P-value | P-value |
| **Determinant** |  |  |  |  |
| Prediabetes versus NGM | 5,455 | NA | NA | 0.07 |
| Type 2 diabetes versus NGM | 5,455 | NA | NA | 0.49 |
| Fasting plasma glucose | 5,454 | 0.81 | 0.85 | 0.69 |
| 2-hour post-load glucose | 5,180 | 0.61 | 0.77 | 0.26 |
| HbA1c | 5,449 | 0.27 | 0.77 | 0.98 |
| Skin autofluorescence | 5,132 | 0.97 | 0.90 | 0.35 |
| Incremental glucose peak | 2,407 | 0.30 | 0.76 | 0.76 |
| CGM-assessed standard deviation | 622 | 0.59 | 0.55 | 0.85 |

P-values represent the P-values for the interaction terms of sex, glucose metabolism status (i.e., prediabetes versus normal glucose metabolism status or type 2 diabetes versus normal glucose metabolism status) with determinants (e.g., sex*HbA1c) in the associations of glucose metabolism status, measures of glycaemia, incremental glucose peak, and CGM-assessed standard deviation with RNFL thickness.

Variables in the model in addition to determinants and interaction term(s) with sex, glucose metabolism status and are: age, sex, educational status, office systolic blood pressure, total cholesterol/HDL cholesterol ratio, use of antihypertensive or lipid-modifying medication, waist circumference, smoking status, and alcohol consumption status. In addition, for interaction analyses with glucose metabolism status, glucose metabolism status was also entered in the model. Additionally, and only for CGM-assessed SD, we entered ‘visit interval’ in model 1.

P value < 0.05 denotes statistically significant interaction.

Abbreviations: CGM, continues glucose monitoring; GMS, glucose metabolism status; HbA1c, hemoglobin A1c; NA, not applicable; NGM, normal glucose metabolism; RNFL, retinal nerve fiber layer.

Supplemental Table S5 Associations of GMS, measures of glycaemia, and indices of daily glucose variability with RNFL thickness after additional adjustment for dietary intake and physical activity (model 4A), for spherical equivalent and intraocular pressure (model 4B), or for eGFR, urinary albumin excretion, history of CVD, plasma biomarkers of low-grade inflammation, retinopathy, and glaucoma (model 4C)

|  |  | | **RNFL thickness, per SD** | |  | |
| --- | --- | --- | --- | --- | --- | --- |
|  | **Model 4A*** | | **Model 4B** | | **Model 4C** | |
|  |  | RNFL, per SD |  | RNFL, per SD |  | RNFL, per SD |
|  | Number of participants | stβ (95% CI) | Number of participants | stβ (95% CI) | Number of participants | stβ (95% CI) |
| **Glucose metabolism status** |  |  |  |  |  |  |
| Prediabetes versus NGM | 4,353 | -0.07 (-0.16; 0.02) | 5,107 | -0.06 (-0.14; 0.02) | 2,148 | -0.08 (-0.20; 0.05) |
| Type 2 diabetes versus NGM | 4,353 | **-0.17 (-0.26; -0.08)** | 5,107 | **-0.15 (-0.23; -0.07)** | 2,148 | **-0.20 (-0.32; -0.07)** |
| **Measures of glycaemia** |  |  |  |  |  |  |
| Fasting plasma glucose, per SD | 4,352 | **-0.04 (-0.07; -0.002)** | 5,106 | **-0.03 (-0.06; -0.002)** | 2,147 | -0.04 (-0.08; 0.01) |
| 2-hour post-load, per SD | 4,130 | **-0.06 (-0.10; -0.02)** | 4,873 | **-0.05 (-0.08; -0.02)** | 2,015 | **-0.07 (-0.12; -0.02)** |
| HbA1c, per SD | 4,348 | **-0.04 (-0.08; -0.01)** | 5,102 | **-0.04 (-0.07; -0.01)** | 2,145 | -0.04 (-0.09; 0.01) |
| Skin autofluorescence, per SD | 4,091 | **-0.04 (-0.08; -0.01)** | 4,805 | **-0.04 (-0.07; -0.01)** | 2,071 | **-0.07 (-0.12; -0.02)** |
| **Indices of daily glucose variability** |  |  |  |  |  |  |
| Incremental glucose peak, per SD | 1,187 | **-0.08 (-0.13; -0.02)** | 2,251 | -0.03 (-0.08; 0.02) | 1,637 | -0.05 (-0.10; 0.01) |
| Model 4 + HbA1c | 1,187 | **-0.08 (-0.15; -0.01)** | 2,251 | -0.03 (-0.09; 0.02) | 1,637 | -0.06 (-0.13; 0.01) |
| CGM- assessed standard deviation, per SD | 439 | -0.09 (-0.20; 0.02) | 615 | -0.06 (-0.15; 0.02) | 592 | -0.06 (-0.15; 0.03)† |
| Model 4 + MSG | 439 | -0.08 (-0.24; 0.08) | 615 | -0.07 (-0.19; 0.06) | 592 | -0.07 (-0.21; 0.07)† |

Standardized regression coefficient (stβ) represents the difference in RNFL thickness in SD for individuals with type 2 diabetes or prediabetes versus NGM or per SD greater measure of glycaemia or daily glucose variability. In the GMS, fasting plasma glucose, 2-hour post-load glucose, HbA1c, and skin autofluorescence study populations 1 SD corresponds with 10.8 μm for RNFL thickness, and (respectively) 1.5 mmol/L for fasting plasma glucose, 4.0 mmol/L for 2-hour post-load glucose, 0.8% or 9.1 mmol/mol for HbA1c, and 0.5 AU for skin autofluorescence (model 4A). For incremental glucose peak, 1 SD corresponds with 11.1 μm for RNFL thickness and 2.9 mmol/L for incremental glucose peak (model 4A). For CGM-assessed standard deviation, 1 SD corresponds with 10.6 μm for RNFL thickness and 0.62 mmol/L for CGM-assessed standard deviation (model 4A). In models 4B and 4C values per SD were numerically comparable. Bold denotes P<0.05.

Variables in model 3: age, sex, educational status, office systolic blood pressure, total cholesterol/HDL cholesterol ratio, use of antihypertensive or lipid-modifying medication, waist circumference, smoking status, and alcohol consumption status. Incremental glucose peak was additionally adjusted for HbA1c. CGM-assessed standard deviation was additionally adjusted for MSG. Additionally, and only for CGM-assessed SD, we entered ‘visit interval’ in model 1.

*Diet intake was entered in the model as diet score minus the alcohol component to avoid multicollinearity.

†Plasma biomarkers of low-grade inflammation were not available in the CGM study population, and were, thus, not included in the analyses.

Abbreviations: AU, arbitrary unit; CGM, continuous glucose monitoring; CI, confidence interval; CVD, cardiovascular disease; eGFR, estimated glomerular filtration rate; GMS, glucose metabolism status; HbA1c, hemoglobin A1c; MSG, mean sensor glucose; NGM, normal glucose metabolism; SD, standard deviation; RNFL, retinal nerve fiber layer.

Supplemental Table S6 Associations of glucose metabolism status, measures of glycaemia, and indices of daily glucose variability with RNFL thickness after exclusion of individuals with retinopathy (model 3A), glaucoma (model 3B), or other types of diabetes (model 3C)

|  |  | | **RNFL thickness, per SD** | |  | |
| --- | --- | --- | --- | --- | --- | --- |
|  | **Model 3A** | | **Model 3B** | | **Model 3C** | |
|  |  | **RNFL, per SD** |  | **RNFL, per SD** |  | **RNFL, per SD** |
|  | Number of participants | stβ (95% CI) | Number of participants | stβ (95% CI) | Number of participants | stβ (95% CI) |
| **Glucose metabolism status** |  |  |  |  |  |  |
| Prediabetes versus NGM | 5,239 | -0.06 (-0.14; 0.02) | 4,902 | -0.02 (-0.11; 0.06) | 5,425 | -0.04 (-0.12; 0.04) |
| Type 2 diabetes versus NGM | 5,239 | **-0.17 (-0.26; -0.09)** | 4,902 | **-0.19 (-0.27; -0.10)** | 5,425 | **-0.16 (-0.24; -0.08)** |
| **Measures of glycaemia** |  |  |  |  |  |  |
| Fasting plasma glucose, per SD | 5,238 | **-0.05 (-0.08; -0.02)** | 4,901 | **-0.05 (-0.08; -0.01)** | 5,424 | **-0.04 (-0.07; -0.01)** |
| 2-hour post-load glucose, per SD | 5,010 | **-0.06 (-0.09; -0.02)** | 4,689 | **-0.06 (-0.10; -0.03)** | 5,180 | **-0.06 (-0.09; -0.02)** |
| HbA1c, per SD | 5,233 | **-0.06 (-0.09; -0.03)** | 4,897 | **-0.05 (-0.08; -0.02)** | 5,419 | **-0.05 (-0.08; -0.02)** |
| Skin autofluorescence, per SD | 4,927 | **-0.05 (-0.08; -0.01)** | 4,616 | **-0.04 (-0.07; -0.01)** | 5,103 | **-0.04 (-0.07; -0.01)** |
| **Indices of daily glucose variability** |  |  |  |  |  |  |
| Incremental glucose peak, per SD | 2,322 | **-0.06 (-0.11; -0.01)** | 1,542 | **-0.05 (-0.11; -0.01)** | 2,407 | **-0.06 (-0.11; -0.01)** |
| Model 3 + HbA1c | 2,322 | **-0.06 (-0.12; -0.01)** | 1,542 | -0.05 (-0.12; 0.02) | 2,407 | **-0.06 (-0.12; -0.01)** |
| CGM- assessed standard deviation, per SD | 594 | -0.08 (-0.18; 0.01) | 564 | -0.07 (-0.17; 0.03) | 620 | -0.08 (-0.17; 0.01) |
| Model 3 + MSG | 594 | -0.06 (-0.20; 0.08) | 564 | -0.07 (-0.22; 0.07) | 620 | -0.06 (-0.19; 0.08) |

Standardized regression coefficient (stβ) represents the difference in RNFL thickness in SD for individuals with type 2 diabetes or prediabetes versus individuals with NGM or per SD greater measure of glycaemia or daily glucose variability. In the GMS, fasting plasma glucose, 2-hour post-load glucose, HbA1c, and skin autofluorescence study populations, 1 SD corresponds with 10.8 μm for RNFL thickness,1.5 mmol/L for fasting plasma glucose, 4.0 mmol/L for 2-hour post-load glucose, 0.8% or 9.1 mmol/mol for HbA1c, and 0.5 AU for skin autofluorescence (model 3A). For incremental glucose peak, 1 SD corresponds with 11.1 μm for RNFL thickness and 2.9 mmol/L for incremental glucose peak (model 3A). For CGM-assessed standard deviation, 1 SD corresponds with 10.8 μm for RNFL thickness and 0.57 mmol/L for CGM-assessed standard deviation (model 3A). In models 3B and 3C values per SD were numerically comparable. Bold denotes P<0.05.

Variables in model 3: age, sex, educational status, office systolic blood pressure, total cholesterol/HDL cholesterol ratio, use of antihypertensive or lipid-modifying medication, waist circumference, smoking status, and alcohol consumption status. Incremental glucose peak was additionally adjusted for HbA1c (model 3 + HbA1c). CGM- assessed standard deviation was additionally adjusted for MSG (model 3 + MSG). Additionally, and only for CGM-assessed SD, we entered ‘visit interval’ in model 1.

Abbreviations: AU, arbitrary unit; CGM, continues glucose monitoring; CI, confidence interval; GMS, glucose metabolism status; HbA1c, hemoglobin A1c; MSG, mean sensor glucose; NGM, normal glucose metabolism; SD, standard deviation; RNFL, retinal nerve fiber layer.

Supplemental Table S7 Associations or OGTT-based indices of daily glucose variability and CGM-based measures with RNFL thickness

|  |  | **RNFL thickness, per SD** | | | |  |
| --- | --- | --- | --- | --- | --- | --- |
|  | Number of participants | Model 1   stβ (95% CI) | Model 2   stβ (95% CI) | Model 3   stβ (95% CI) | Model 3 + HbA1c  stβ (95% CI) | Model 3 + SD  stβ (95% CI) |
| **Other OGTT-based measures** |  |  |  |  |  |  |
| Maximum glucose peak, per SD | 2,407 | **-0.09 (-0.13; -0.05)** | **-0.07 (-0.11; -0.03)** | **-0.05 (-0.10; -0.01)** | -0.06 (-0.12; 0.004) | NA |
| 1-hour post-load glucose, per SD | 2,531 | **-0.07 (-0.11; -0.03)** | **-0.05 (-0.10; -0.01)** | -0.04 (-0.08; 0.01) | -0.03 (-0.09; 0.03) | NA |
| **CGM-based measures** |  |  |  |  |  |  |
| Mean sensor glucose, per SD | 622 | **-0.10 (-0.18; -0.01)** | -0.08 (-0.17; 0.004) | -0.07 (-0.17, 0.03) | NA | -0.02 (-0.16; 0.13) |
| Coefficient of variation, per SD | 622 | -0.07 (-0.15; 0.01) | -0.07 (-0.15; 0.02) | -0.06 (-0.15; 0.02) | NA | NA |
| Time in range, per SD | 622 | 0.06 (-0.03; 0.14) | 0.05 (-0.04; 0.13) | 0.03 (-0.06; 0.12) | NA | NA |
| Time below range, per SD | 622 | -0.03 (-0.11; 0.05) | -0.03 (-0.11; 0.05) | -0.02 (-0.10; 0.05) | NA | NA |
| Time above range, per SD | 622 | -0.03 (-0.11; 0.05) | -0.03 (-0.10; 0.06) | -0.01 (-0.10; 0.08) | NA | NA |

Results (β [95% confidence interval]) represent the difference in retinal nerve fiber layer thickness (in SD) for one SD greater exposure to a determinant. For incremental glucose peak and maximum glucose peak, 1 SD corresponds with 11.1 μm for RNFL thickness, 2.9 mmol/L for incremental glucose peak and 3.9 mmol/L for maximum glucose peak. For 1-hour post-load glucose, 1 SD corresponds with 11.0 μm for RNFL thickness and 4.0 mmol/L for 1-hour post-load glucose. For the CGM-based measures, 1 SD corresponds with 10.8 μm for RNFL thickness, 1.3 mmol/L for mean sensor glucose, 5.6% for coefficient of variation, 12.9% for time in range, 2.5% time below range, and12.7X% time above range.

Model 1 was not adjusted for potential confounders (crude); model 2 was adjusted for age, sex, and education level [low, middle, high]; model 3 was additionally adjusted for waist circumference, office systolic blood pressure, antihypertensive medication, total cholesterol to HDL cholesterol ratio, use of lipid-modifying medication, smoking status [current, ever, never], and alcohol consumption status [none, low, high]; model 3 + HbA1c was additionally adjusted for HbA1c (in case of maximum glucose peak, 1-hour post-load glucose). Model 3 + SD was additionally adjusted for CGM-assessed standard deviation (only applicable for mean sensor glucose). Additionally, and only for CGM-assessed indices, we entered ‘visit interval’ in model 1.

Abbreviations: AU, arbitrary unit; CGM, continues glucose monitoring; CI, confidence interval; GMS, glucose metabolism status; HbA1c, hemoglobin A1c; NGM, normal glucose metabolism; SD, standard deviation; RNFL, retinal nerve fiber layer.

Supplemental Table S8 Associations of glucose metabolism status, measures of glycaemia, and indices of daily glucose variability with RNFL thickness, where waist circumference was replaced with BMI (model 3A) or educational level was replaced with occupational status (model 3B) or income level (model 3C)

|  |  | | **RNFL thickness, per SD** | |  | |
| --- | --- | --- | --- | --- | --- | --- |
|  | **Model 3A** | | **Model 3B** | | **Model 3C** | |
|  |  | RNFL, per SD |  | RNFL, per SD |  | RNFL, per SD |
|  | Number of participants | stβ (95% CI) | Number of participants | stβ (95% CI) | Number of participants | stβ (95% CI) |
| **Glucose metabolism status** |  |  |  |  |  |  |
| Prediabetes versus NGM | 5,456 | -0.05 (-0.13; 0.03) | 1,847 | -0.10 (-0.23; 0.04) | 4,189 | -0.06 (-0.15; 0.04) |
| Type 2 diabetes versus NGM | 5,456 | **-0.17 (-0.25; -0.09)** | 1,847 | **-0.20 (-0.34; -0.06)** | 4,189 | **-0.16 (-0.26; -0.07)** |
| **Measures of glycaemia** |  |  |  |  |  |  |
| Fasting plasma glucose, per SD | 5,455 | **-0.05 (-0.08; -0.02)** | 1,847 | -0.04 (-0.09; 0.01) | 4,188 | **-0.05 (-0.08; -0.01)** |
| 2-hour post-load glucose , per SD | 5,181 | **-0.06 (-0.09; -0.03)** | 1,727 | **-0.08 (-0.14; -0.03)** | 3,998 | **-0.05 (-0.09; -0.01)** |
| HbA1c, per SD | 5,450 | **-0.05 (-0.09; -0.02)** | 1,843 | -0.04 (-0.09; 0.02) | 4,184 | **-0.05 (-0.08; -0.01)** |
| Skin autofluorescence, per SD | 5,133 | **-0.04 (-0.07; -0.01)** | 1,781 | **-0.06 (-0.11; -0.01)** | 3,942 | **-0.05 (-0.08; -0.01)** |
| **Indices of daily glucose variability** |  |  |  |  |  |  |
| Incremental glucose peak, per SD | 2,407 | **-0.06 (-0.11; -0.02)** | 1,541 | -0.06 (-0.11; 0.003) | 1,860 | -0.05 (-0.10; 0.004) |
| Model 3 + HbA1c | 2,407 | **-0.06 (-0.12; -0.01)** | 1,541 | -0.06 (-0.13; 0.01) | 1,860 | -0.04 (-0.11; 0.02) |
| CGM- assessed standard deviation, per SD | 622 | -0.08 (-0.17; 0.01) | 0 | N/A | 483 | -**0.13 (-0.13; -0.02)** |
| Model 3 + MSG | 622 | -0.07 (-0.21; 0.07) | 0 | N/A | 483 | -0.11 (-0.27; 0.05) |

Standardized regression coefficient (stβ) represents the difference in RNFL thickness in SD for individuals with type 2 diabetes or prediabetes versus individuals with NGM or per SD greater measure of glycaemia or daily glucose variability. In the GMS, fasting plasma glucose, 2-hour post-load glucose, HbA1c, and skin autofluorescence study populations, 1 SD corresponds with 10.8 μm for RNFL thickness, 1.5 mmol/L for fasting plasma glucose, 4.0 mmol/L for 2-hour post-load glucose, 0.8% or 9.1 mmol/mol for HbA1c, and 0.5 AU for skin autofluorescence (model 3A). For incremental glucose peak, 1 SD corresponds with 11.1 μm for RNFL thickness and 2.9 mmol/L for incremental glucose peak (model 3A). For CGM-assessed standard deviation, 1 SD corresponds with 10.8 μm for RNFL thickness and 0.58 mmol/L for CGM-assessed standard deviation (model 3A). In models 3B and 3C values per SD were numerically comparable.

Bold denotes P<0.05.

Variables in model 3: age, sex, educational status (where applicable), office systolic blood pressure, total cholesterol/HDL cholesterol ratio, use of antihypertensive or lipid-modifying medication, waist circumference (where applicable), smoking status, and alcohol consumption status. Incremental glucose peak was additionally adjusted for HbA1c (model 3+ HbA1c). CGM-assessed standard deviation was additionally adjusted for MSG. Additionally, and only for CGM-assessed SD, we entered ‘visit interval’ in model 1.

Abbreviations: AU, arbitrary unit; BMI, body mass index; CGM, continues glucose monitoring; CI, confidence interval; GMS, glucose metabolism status; HbA1c, hemoglobin A1c; MSG, mean sensor glucose; NGM, normal glucose metabolism; SD, standard deviation; RNFL, retinal nerve fiber layer.

Supplemental Table S9 Associations of glucose metabolism status, measures of glycaemia, and indices of daily glucose variability with RNFL thickness where office systolic blood pressure was replaced with office diastolic blood pressure (model 3A), 24-hour ambulatory systolic blood pressure (model 3B), or 24-hour ambulatory diastolic blood pressure (model 3C)

|  |  | | **RNFL thickness, per SD** | |  | |
| --- | --- | --- | --- | --- | --- | --- |
|  | **Model 3A** | | **Model 3B** | | **Model 3C** | |
|  |  | RNFL, per SD |  | RNFL, per SD |  | RNFL, per SD |
|  | Number of participants | stβ (95% CI) | Number of participants | stβ (95% CI) | Number of participants | stβ (95% CI) |
| **Glucose metabolism status** |  |  |  |  |  |  |
| Prediabetes versus NGM | 5,454 | -0.05 (-0.13; 0.03) | 4,921 | -0.04 (-0.13; 0.04) | 4,921 | -0.04 (-0.13; 0.04) |
| Type 2 diabetes versus NGM | 5,454 | **-0.17 (-0.25; -0.09)** | 4,921 | **-0.16 (-0.25; -0.08)** | 4,921 | **-0.17 (-0.25; -0.08)** |
| **Measures of glycaemia** |  |  |  |  |  |  |
| Fasting plasma glucose, per SD | 5,453 | **-0.05 (-0.08; -0.02)** | 4,920 | **-0.05 (-0.08; -0.02)** | 4,920 | **-0.05 (-0.08; -0.02)** |
| 2-hour post-load glucose , per SD | 5,179 | **-0.06 (-0.09; -0.02)** | 4,683 | **-0.05 (-0.09; -0.02)** | 4,683 | **-0.05 (-0.09; -0.02)** |
| HbA1c, per SD | 5,448 | **-0.05 (-0.08; -0.02)** | 4,915 | **-0.05 (-0.08; -0.02)** | 4,915 | **-0.05 (-0.08; -0.02)** |
| Skin autofluorescence, per SD | 5,131 | **-0.04 (-0.08; -0.01)** | 4,627 | **-0.04 (-0.07; -0.01)** | 4,627 | **-0.04 (-0.07; -0.01)** |
| **Indices of daily glucose variability** |  |  |  |  |  |  |
| Incremental glucose peak, per SD | 2,407 | **-0.06 (-0.10; -0.01)** | 2,134 | -0.05 (-0.10; 0.002) | 2,134 | -0.05 (-0.10; 0.001) |
| Model 3 + HbA1c | 2,407 | **-0.06 (-0.12; -0.01)** | 2,134 | -0.06 (-0.12; 0.003) | 2,134 | -0.06 (-0.12; 0.001) |
| CGM- assessed standard deviation, per SD | 622 | -0.08 (-0.17; 0.01) | 562 | -0.08 (-0.17; 0.02) | 562 | -0.07 (-0.17; 0.02) |
| Model 3 + MSG | 622 | -0.07 (-0.21; 0.07) | 562 | -0.08 (-0.22; 0.06) | 562 | -0.08 (-0.22; 0.06) |

Standardized regression coefficient (stβ) represents the difference in RNFL thickness in SD for individuals with type 2 diabetes or prediabetes versus individuals with NGM or per SD greater measure of glycaemia or daily glucose variability. In the GMS, fasting plasma glucose, 2-hour post-load glucose, HbA1c, and skin autofluorescence study populations, 1 SD corresponds with 10.8 μm for RNFL thickness, 1.5 mmol/L for fasting plasma glucose, 4.0 mmol/L for 2-hour post-load glucose, 0.8% or 9.1 mmol/mol for HbA1c, and 0.5 AU for skin autofluorescence (model 3A). For incremental glucose peak, 1 SD corresponds with 11.1 μm for RNFL thickness and 2.9 mmol/L for incremental glucose peak (model 3A). For CGM-assessed standard deviation, 1 SD corresponds with 10.8 μm for RNFL thickness and 0.58 mmol/L for CGM-assessed standard deviation (model 3A). In models 3B and 3C values per SD were numerically comparable. Bold denotes P<0.05.

Variables in model 3: age, sex, educational status, office diastolic blood pressure (where applicable), total cholesterol/HDL cholesterol ratio, use of antihypertensive or lipid-modifying medication, waist circumference (where applicable), smoking status, and alcohol consumption status. Incremental glucose peak was additionally adjusted for HbA1c (model 3+ HbA1c). CGM-assessed standard deviation was additionally adjusted for MSG. Additionally, and only for CGM-assessed SD, we entered ‘visit interval’ in model 1.

Abbreviations: CGM, continues glucose monitoring; CI, confidence interval; GMS, glucose metabolism status; HbA1c, hemoglobin A1c; MSG, mean sensor glucose; NGM, normal glucose metabolism; SD, standard deviation; RNFL, retinal nerve fiber layer.

Supplemental Table S10 Standardized regression coefficients of skin autofluorescence with retinal nerve fiber thickness after additional adjustment for fasting plasma glucose, 2-hour post-load glucose, or HbA1c

|  |  | | **RNFL thickness, per SD** | |  | |  | |  | |
| --- | --- | --- | --- | --- | --- | --- | --- | --- | --- | --- |
|  | **Model 3 + fasting plasma glucose** | | **Model 3 + 2-hour post-load glucose** | | | **Model 3 + HbA1c** | | | |  |
|  | Number of  participants | stβ (95% CI) | Number of participants | stβ (95% CI) | | Number of participants | | stβ (95% CI) | |  |
| SAF, per SD | 5,131 | **-0.04 (-0.07; -0.01)** | 4,872 | **-0.04 (-0.07; -0.01)** | | 5,126 | | **-0.04 (-0.07; -0.01)** | |  |

Results (β [95% confidence interval]) represent the difference in retinal nerve fiber layer thickness (in SD) for one SD greater exposure to a determinant. One SD corresponds with 10.9 μm for the RNFL and 0.5 AU for SAF in model 3+fasting plasma glucose. In models 3B and 3C values per SD were numerically comparable.

Bold denotes P<0.05.

The associations were adjusted for age, sex, education level [low, middle, high], waist circumference, office systolic blood pressure, antihypertensive medication, total cholesterol to HDL cholesterol ratio, use of lipid-modifying medication, smoking status [current, ever, never], alcohol consumption status [none, low, high], and fasting plasma glucose (model 3 + fasting plasma glucose), 2-hour post load glucose (model 3 + 2-hour post load glucose), or HbA1c (model 3 + HbA1c).

Abbreviations: SAF, skin autofluorescence; CI, confidence interval; HbA1c, hemoglobin A1c; SD, standard deviation; RNFL, retinal nerve fiber layer; SAF, skin autofluorescence

Supplemental Table S11 Standardized regression coefficients of incremental glucose peak and CGM-assessed standard deviation with retinal nerve fiber thickness after replacement of HbA1c with fasting plasma glucose or skin autofluorescence, or mean sensor glucose with fasting plasma glucose, skin autofluorescence, or HbA1c

|  | **Retinal nerve fiber layer thickness, per SD** | |  | |  | |  | |  | |
| --- | --- | --- | --- | --- | --- | --- | --- | --- | --- | --- |
|  | **Model 3 + fasting plasma glucose** | | **Model 3 + SAF** | | | **Model 3 + HbA1c** | | | |  |
|  | Number of  participants | stβ (95% CI) | Number of participants | stβ (95% CI) | | Number of participants | | stβ (95% CI) | |  |
| Incremental glucose peak, per SD | 2,407 | **-0.07 (-0.13; -0.01)** | 2,295 | **-0.05 (-0.10; -0.01)** | | N/A | | N/A | |  |
| CGM-assessed standard deviation, per SD | 622 | -0.06 (-0.17; 0.04) | 573 | -0.07 (-0.17; 0.03) | | 622 | | -0.05 (-0.16; 0.07) | |  |

Results (β [95% confidence interval]) represent the difference in retinal nerve fiber layer thickness (in SD) for 1 SD greater exposure to a determinant. For the incremental glucose peak results, 1 SD corresponds with 11.1 μm for the RNFL and 2.9 mmol/L for incremental glucose peak. For the CGM-assessed standard deviation results, 1 SD corresponds with 10.7 μm for the RNFL and 0.58 mmol/L for CGM-assessed standard deviation.

The associations were adjusted for age, sex, education level [low, middle, high], waist circumference, office systolic blood pressure, antihypertensive medication, total cholesterol to HDL cholesterol ratio, use of lipid-modifying medication, smoking status [current, ever, never], alcohol consumption status [none, low, high], and fasting plasma glucose (model 3 + fasting plasma glucose), skin autofluorescence (model 3 + SAF), or HbA1c (model 3 + HbA1c). Additionally, and only for CGM-assessed SD, we entered ‘visit interval’ in model 1.

Abbreviations: CGM, continues glucose monitoring; CI, confidence interval; HbA1c, hemoglobin A1c; SD, standard deviation; RNFL, retinal nerve fiber layer; SAF, skin autofluorescence

Supplemental Table S12 Associations of continuous glucose monitoring-assessed standard deviation with retinal nerve fiber thickness after exclusion of individuals with less than 3 days of CGM data available (model 3A), individuals with CGM data gaps (model 3B), or with a ‘visit interval’ (Model 3C)

|  |  | |  | **RNFL thickness, per SD** |  |  |
| --- | --- | --- | --- | --- | --- | --- |
|  | **Model 3A** | | **Model 3B** | | **Model 3C*** | |
|  | Number of participants | stβ (95% CI) | Number of participants | stβ (95% CI) | Number of participants | stβ (95% CI) |
| CGM-assessed standard deviation, per SD | 596 | -0.09 (-0.18; 0.01) | 573 | **-0.10 (-0.19; -0.001)** | 409 | 0.01 (-0.10; 0.12) |
| Model 3 + MSG | 596 | -0.08 (-0.23; 0.06) | 573 | -0.08 (-0.23; 0.07) | 409 | 0.02 (-0.12; 0.17) |

Results (β [95% confidence interval]) represent the difference in retinal nerve fiber layer thickness (in SD) for 1 SD greater exposure to a determinant. For the results, 1 SD corresponds with 10.7μm for the RNFL and 0.58 mmol/L for CGM-assessed standard deviation (model 3A). In models 3B and 3C values per SD were numerically comparable. Bold denotes P<0.05.

The associations were adjusted for visit interval, age, sex, education level [low, middle, high], waist circumference, office systolic blood pressure, antihypertensive medication, total cholesterol to HDL cholesterol ratio, use of lipid-modifying medication, smoking status [current, ever, never], alcohol consumption status [none, low, high], and mean sensor glucose.

* Of note, model 3C was not adjusted for visit interval, as all individuals had an interval of 0 years.

Abbreviations: CGM, continues glucose monitoring; CI, confidence interval; GMS, glucose metabolism status; MSG, mean sensor glucose; SD, standard deviation; RNFL, retinal nerve fiber layer.

Supplemental Table S13 Associations of CGM-assessed standard deviation and mean sensor glucose with retinal nerve fiber thickness estimated with ridge regression and presented for different degrees of penalization

|  |  | **RNFL thickness , per SD** |  |
| --- | --- | --- | --- |
| VIF | Number of participants | CGM-assessed standard deviation, per SD  (st.β, 95% CI) | CGM-assessed mean sensor glucose, per SD  (st.β, 95% CI) |
| Model 3 + MSG (λ=0) | 622 | -0.063 (-0.183; 0.058) | -0.021 (-0.158; 0.121) |
| Halfway (λ=0.04) | 622 | -0.062 (-0.183; 0.056) | -0.023 (-0.156; 0.114) |
| Model 3 (λ=0.10) | 622 | -0.063 (-0.186; 0.053) | -0.020 (-0.152; 0.103) |

Standardized regression coefficients (st.β) indicate the median difference (95% confidence interval) associated with 1 SD higher SD or MSG. All coefficients were estimated by use of ridge regression. We pragmatically chose the level of penalization based on the λ required to reduce the variance inflation factor (VIF) of model 3 + MSG back to the VIF of model 3 (or halfway back). Point estimates and 95% confidence intervals were calculated by use of 1,000 bootstraps estimates.

The associations were adjusted for visit interval, age, sex, education level [low, middle, high], waist circumference, office systolic blood pressure, antihypertensive medication, total cholesterol to HDL cholesterol ratio, use of lipid-modifying medication, smoking status [current, ever, never], alcohol consumption status [none, low, high], and mean sensor glucose.

Abbreviations: CGM, continues glucose monitoring; CI, confidence interval; GMS, glucose metabolism status; MSG, mean sensor glucose; SD, standard deviation; RNFL, retinal nerve fiber layer.

Supplemental S14 Associations of duration of diabetes and age with retinal nerve fiber layer thickness

|  |  |  | **RNFL thickness, per SD** |  |
| --- | --- | --- | --- | --- |
|  |  | **Model 1** | **Model 2** | **Model 3** |
|  | Number of participants | stβ (95% CI) | stβ (95% CI) | stβ (95% CI) |
| Duration of diabetes, per SD | 982 | **-0.07 (-0.13; -0.004)** | -0.06 (-0.13; 0.001) | **-0.07 (-0.13; -0.001)** |
| Age, per SD | 5,180 | **-0.06 (-0.08; -0.03)** | **-0.11 (-0.13; -0.08)** | **-0.09 (-0.12; -0.06)** |

Standardized regression coefficient (stβ) represents the difference in RNFL thickness in SD per SD longer duration of diabetes or age. One SD corresponds with 7.4 years for duration of diabetes or 8.7 years for age and 11.2 μm or 10.9 μm for RNFL thickness in respectively the duration of diabetes study population and the age study population.

Bold denotes P<0.05.

Variables entered in the models in addition to duration of diabetes or age: model 1: none (crude results); model 2: age (where applicable), sex, and educational status (low, medium, high); model 3: model 2 + office systolic blood pressure, total cholesterol to HDL cholesterol ratio, use of antihypertensive or lipid-modifying medication (yes/no), waist circumference, smoking status (current, ever, never), and alcohol consumption status (none, low, high). Additionally, and only for analyses with age as determinant, glucose metabolism status and spherical equivalent were entered in model 2.

We compared the standardized betas of type 2 diabetes versus NGM (i.e. -0.16SD) with the standardized beta for age to calculate that beta of type 2 diabetes [stβ, -0.16] corresponds with approximately 15 years of aging (calculated as stβ_type 2 diabetes_/ stβ_age_ * number of years per SD = [0.16/0.09]* 8.7 years = 15 years).

Abbreviations: CI, confidence interval; HDL, high-density lipoprotein; SD, standard deviation; RNFL, retinal nerve fiber layer; NGM, normal glucose metabolism; FPG, fasting plasma glucose; HbA1c, hemoglobin A1c.
